# Efficacy and safety of intralymphatic immunotherapy in allergic rhinitis: A systematic review and meta‐analysis

**DOI:** 10.1002/clt2.12055

**Published:** 2021-08-17

**Authors:** Nor Rahimah Aini, Norhayati Mohd Noor, Mohd Khairi Md Daud, Sarah K. Wise, Baharudin Abdullah

**Affiliations:** ^1^ Department of Otorhinolaryngology‐Head and Neck Surgery School of Medical Sciences Universiti Sains Malaysia Health Campus Kelantan Malaysia; ^2^ Department of Family Medicine School of Medical Sciences Universiti Sains Malaysia Kelantan Malaysia; ^3^ Department of Otolaryngology‐Head & Neck Surgery Emory University School of Medicine Atlanta Georgia USA

**Keywords:** allergen‐specific immunotherapy, allergic rhinitis, efficacy, intralymphatic immunotherapy, safety

## Abstract

**Background:**

Intralymphatic immunotherapy (ILIT) is a potential treatment option for allergic rhinitis (AR). We aimed to determine the efficacy (primary outcomes) and safety (secondary outcomes) of ILIT in treating patients with AR.

**Methods:**

An electronic literature search was performed using MEDLINE and Cochrane Central Register of Controlled Trials CENTRAL (from their inception to December 2020). A random‐effects model was used to estimate the pooled prevalence with 95% confidence intervals. This study is registered with PROSPERO (CRD42019126271).

**Results:**

We retrieved a total of 285 articles, of which 11 satisfied our inclusion criteria. There were 452 participants with age ranged from 15 to 58 years old. Intralymphatic immunotherapy was given in three doses with intervals of four weeks between doses in 10 trials. One trial gave three and six doses with an interval of two weeks. Both primary and secondary outcomes showed no difference between ILIT and placebo for all trials. There was no difference in the combined symptoms and medication score (SMD ‐0.51, 95% CI −1.31 to 0.28), symptoms score (SMD −0.27, 95% CI −0.91 to 0.38), medication score (SMD −6.56, 95% CI −21.48 to 8.37), rescue medication (RR 12.32, 95% CI 0.72–211.79) and the overall improvement score (MD −0.07, 95% CI −2.28 to 2.14) between ILIT and placebo. No major adverse events noted.

**Conclusions:**

Intralymphatic immunotherapy possibly has a role in the treatment of AR patients. This review found it is safe but not effective, which could be contributed by the high variation amongst the trials. Future trials should involve larger numbers of participants and report standardized administration of ILIT and outcome measures.

## 1 BACKGROUND

The prevalence of self‐reported allergic rhinitis (AR) has been estimated to be approximately 25% in children and more than 40% in adults.^1^ Allergic rhinitis is frequently associated with asthma which is found in 15%–38% of AR patients while nasal symptoms present in 6%–85% of patients with asthma.^2^ The management of AR involves patient education, allergen (and pollutant) avoidance, pharmacotherapy, and allergen‐specific immunotherapy (AIT) when appropriate.^3^ Pharmacologic options for the treatment of AR include intranasal corticosteroids, oral and topical antihistamines, decongestants, intranasal cromolyn, intranasal anticholinergics and leukotriene receptor antagonist, amongst others.^4^, ^5^ A recent advance has been the combination of an antihistamine and a corticosteroid in the same nasal spray.^3^

Allergen‐specific immunotherapy should be considered for patients with moderate or severe persistent AR that is not responsive to pharmacological treatments.^6^ The most widely employed form of AIT involves the administration, subcutaneously or sublingually, of increasing doses of the causative allergen to induce clinical and immunologic tolerance. However, the conventional subcutaneous immunotherapy (SCIT) requires 30 to 80 allergen injections over three to five years and maybe associated with allergic side effects.^3^, ^6^, ^7^ Sublingual immunotherapy (SLIT) is more patient‐friendly, but treatment duration could not be shortened.^8^ Intralymphatic immunotherapy (ILIT) is a potential treatment option to overcome these limitations.

Intralymphatic immunotherapy is a form of AIT where the allergen is directly delivered to B‐ and T‐cells within the lymph nodes. This procedure is reported to induce a stronger cytotoxic T‐cell response and higher immunogenicity than other routes, even with the delivery of smaller amounts of allergen as typically employed in ILIT.^9^ Allergenic extract is usually injected into inguinal lymph nodes using ultrasound guidance. Therapy is complete after only three injections. When given four weeks apart, ILIT is reported to be safe and effective for up to three years.^10^, ^11^, ^12^, ^13^ In patients with allergic rhinitis that require immunotherapy treatment, ILIT might be an option to treat their symptoms. This meta‐analysis aimed to evaluate the efficacy and safety of ILIT in the treatment of AR.

## 2 METHODS

A systematic review and meta‐analysis were undertaken in accordance with the preferred reporting items for systematic reviews and meta‐analyses statement (PRISMA) guideline.^14^ This study is registered with PROSPERO, number CRD42019126271. The search was conducted for randomized clinical trials (RCTs) and case‐control studies comparing ILIT with placebo or conventional AIT in patients diagnosed with AR (with or without allergic conjunctivitis or urticaria or asthma). The search was not limited by age, gender, race, or ethnicity. Allergic rhinitis required clinician diagnosis. Types of interventions included AIT with single or multiple allergens involving recombinant, synthetic or natural allergens. The principle of the Grades of Recommendation, Assessment, Development and Evaluation (GRADE) approach for evaluating the quality of evidence was used.^15^

The search was conducted using the following MeSH terms “allergic rhinitis” OR “rhinoconjunctivitis” AND “intralymphatic immunotherapy” OR “intralymphatic allergen” AND “sublingual immunotherapy” OR “subcutaneous immunotherapy” in MEDLINE (1966 to Dec 2020) and Cochrane Central Register of Controlled Trials CENTRAL (Dec 2020) (Table 1). Reference lists of identified RCTs and review articles were evaluated to find unpublished trials or trials not identified by electronic searches. Finally, ongoing trials were searched through the World Health Organization WHO International Clinical Trials Registry Platform (http://www.who.int/ictrp/en/) and www.clinicaltrials.gov.

**TABLE 1:** Search strategy

Databases	Search strategy
PubMed	(Allergic rhinitis [Title/Abstract]) OR (rhinoconjunctivitis [Title/Abstract]) AND (intralymphatic immunotherapy [Title/Abstract]) OR (intralymphatic allergen [Title/Abstract]) AND (subcutaneous immunotherapy [Title/Abstract]) AND (randomized controlled trial [Filter]) OR (sublingual immunotherapy [Title/Abstract])
Cochrane	Allergic rhinitis in title abstract keyword AND intralymphatic immunotherapy in title abstract keyword

Two review authors (NRA, BA) screened the titles and abstracts independently from the searches and obtained full‐text articles when they appeared to meet the eligibility criteria or when there was insufficient information to assess the eligibility. The eligibility of the trials was assessed independently and the reasons for exclusion were documented. Any disagreements between the review authors were resolved by discussion. The study authors were contacted when any clarification was required.

Using data extraction form, the study setting, participant characteristics (age, sex, ethnic), methodology (number of participants randomized and analyzed, duration of follow‐up), type of allergen used, method of diagnosing AR, perennial or seasonal group, type of comparison group which was either a placebo or conventional AIT, assessment of duration, technique and number of injection, the occurrence of related adverse events such as local swelling or systemic symptoms such as fatigue and at last the requirement of rescue medication were extracted. The effects of ILIT were assessed at two weeks following completion of treatment with three doses of ILIT. The primary outcomes were combined symptoms medication score, symptoms score, medication score, rescue medication and overall symptoms improvement. The secondary outcomes were adverse event reports (i.e., anaphylaxis or other systemic reaction, reactivation of herpes zoster), specific IgE antibody level, allergen sensitivity measures via wheal change after skin prick test and quality of life measures. The risk of bias assessment was based on random sequence generation, allocation concealment, blinding of participants and personnel, blinding of outcome assessors, completeness of outcome data, the selectivity of outcome reporting and other bias.^16^ Any disagreements were resolved by discussion.

The meta‐analyses were done using Review Manager 5.3.5 software.^17^ The statistical analyses were performed using the random‐effects model and the results expressed as risk ratio (RR) for dichotomous outcomes and mean difference (MD) for continuous outcomes with 95% confidence intervals (CI). The included trials were checked for unit of analysis errors. If any cluster‐RCTs were encountered, the results from trials were adjusted to show a unit of analysis errors based on mean cluster size and intracluster correlation coefficient.^16^ If studies have non‐extractable or missing data, analyses of the available data were performed. The presence of heterogeneity was assessed in two steps. First, obvious heterogeneity at face value was assessed by comparing populations, settings, interventions and outcomes. Second, statistical heterogeneity was assessed through the I^2^ statistic.^16^ The heterogeneity was interpreted as outlined: 0% to 40% might not be important; 30% to 60% may represent moderate heterogeneity; 50% to 90% may represent substantial heterogeneity, and 75% to 100% would be considerable heterogeneity.^16^

## 3 RESULTS

**FIGURE 1:**
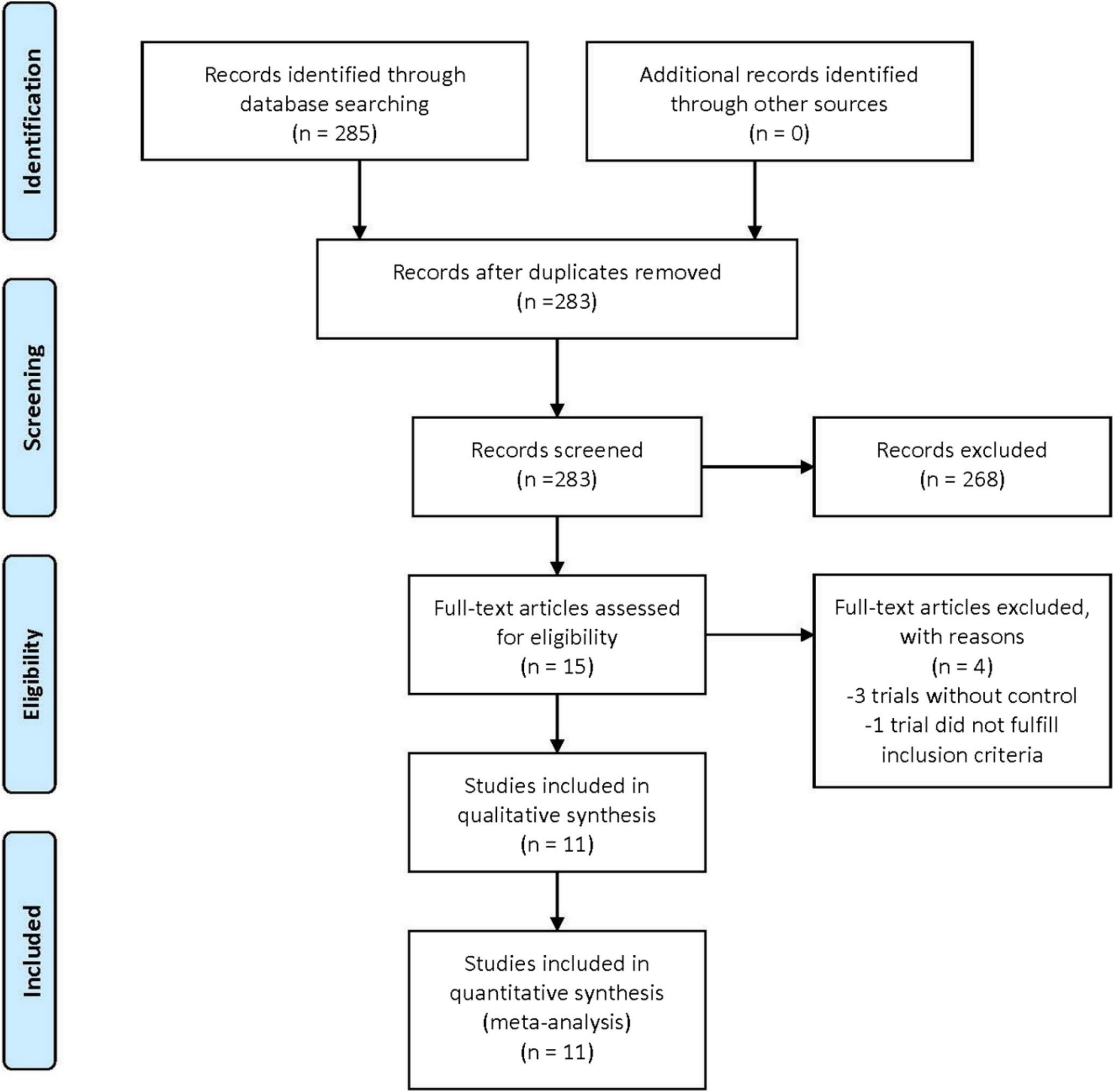
Study flow diagram

A total of 285 records were retrieved and screened from the search of electronic databases (Figure 1). Fifteen full‐text articles were assessed for eligibility and four records were excluded. Three trials^19^, ^20^, ^21^ excluded from the review due to no control group while one trial^22^ did not meet the inclusion criteria. 11 articles^10^, ^11^, ^12^, ^13^, ^18^, ^23^, ^24^, ^25^, ^26^, ^27^, ^28^ were included in the review. The characteristics of the included trials are shown in Table 2.

**TABLE 2:** Characteristics of the included trials

First author, year	Study design	No of patients/ No of trial sites	Diagnosis	Intervention	Control	Injection interval
Senti 2008	RCT	165/1	Patients with seasonal rhinoconjunctivitis	Grass pollen	Grass pollen vaccine via subcutaneous injection	4 weeks
Senti 2011	RCT	20/1	Patients with cat dander allergy	MAT‐ Fel d 1 (cat dander modified recombinant allergen)	Placebo via intralymphatic injection	28 ± 3 days
Hylander 2013	RCT	15/1	Patients with birch‐ pollen or grass‐ pollen rhinoconjunctivitis	Birch or grass pollen	Placebo via intralymphatic injection	4 weeks
Hylander 2013	Case control	13/1	Patients with birch‐ pollen or grass‐ pollen rhinoconjunctivitis	Birch or grass pollen	Birch‐pollen vaccine via subcutaneous injection	4 weeks
Witten 2013	RCT	45/1	Patients with grass pollen rhinoconjunctivitis	Grass pollen	Placebo via intralymphatic injection	14 days
Hylander 2016	RCT	36/1	Patients with birch or grass pollen rhinoconjunctivitis	Birch or grass pollen	Placebo via intralymphatic injection	3 to 4 weeks
Patterson 2016	RCT	15/1	Patients with allergy to grass pollen	Grass pollen	Placebo via intralymphatic injection	4 weeks
Hellkvist 2018	RCT	60/2	Patients with both birch and grass rhinoconjunctivitis	Grass and birch pollen	Placebo via intralymphatic injection	4 weeks
Konradsen 2019	RCT	30/1	Patients with birch and/or timothy grass pollen‐ rhinoconjunctivitis	Birch or grass pollen	Placebo via intralymphatic injection	4 weeks
Thompson 2020	RCT	21/3	Patients with mountain cedar allergy	Mountain cedar pollen	Placebo via intralymphatic injection	4 weeks
Terada 2020	RCT	18/1	Patients with severe Japanese cedar pollen allergic rhinitis	Japanese cedar pollen	Placebo via intralymphatic injection	4 weeks
Skaarup 2020	RCT	36/1	Patients with grass pollen rhinoconjunctivitis	Grass pollen	Placebo via intralymphatic injection (1 or 3 placebo)	4 weeks

Abbreviation: RCT, randomized controlled trial.

### 3.1 Participants

A total of 452 participants were enrolled in the included trials. Nine trials^10^, ^11^, ^12^, ^13^, ^23^, ^24^, ^26^, ^27^, ^28^ involved patients 18 years old and older. Two trials^10^, ^23^ by the same investigators have duplicates participants; a succeeding trial^23^ included 15 participants from an initial study (first cohort).^10^ Thus, the former trial analysis was based on the second cohort of patients with 21 participants. Two trials^18^, ^25^ reported a younger age group from 15 years old. 10 trials^10^, ^11^, ^13^, ^23^, ^24^, ^25^, ^26^, ^27^, ^28^ involving 369 participants mentioned the gender of the participants with a male to female ratio of 1.5:1. Eight trials^10^, ^11^, ^12^, ^23^, ^24^, ^26^, ^27^, ^28^ reported exclusion of participants due to pregnancy or nursing, planning for pregnancy, autoimmune disease, perennial asthma or any other cardiopulmonary disease. In contrast, one trial^25^ includes mild young adult asthma as an inclusion criterion.

### 3.2 Intervention

Participants in the trials were randomized into intervention and control groups. Seven trials^11^, ^12^, ^13^, ^18^, ^26^, ^27^, ^28^ used a single allergen as the ILIT intervention. Four trials^10^, ^23^, ^24^, ^25^ used two different allergens, but only one trial^24^ assessed the result of two different allergens individually. Grass pollen extract was administered in eight trials,^10^, ^11^, ^13^, ^18^, ^23^, ^24^, ^25^, ^26^ birch pollen extract in four trials,^10^, ^23^, ^24^, ^25^ cedar pollen extract was administrated in two trials^27^, ^28^ and one trial^12^ used cat dander allergen extract (MAT‐Fel d 1).

The allergen was administered via superficial inguinal lymph nodes using ultrasound guidance in all 11 trials.^10^, ^11^, ^12^, ^13^, ^18^, ^23^, ^24^, ^25^, ^26^, ^27^, ^28^ In 10 trials,^10^, ^11^, ^12^, ^18^, ^23^, ^24^, ^25^, ^26^, ^27^, ^28^ ILIT was given in three doses with intervals of four weeks between doses. One trial^13^ administered three and six doses with an interval of two weeks. The same dose of allergen was administered in nine trials. Eight trials^10^, ^11^, ^13^, ^23^, ^24^, ^25^, ^26^, ^27^ administered 0.1 ml of 1000 SQ‐U each while one trial^28^ used 0.1 ml of 20 Japanese Allergy Units (JAU). In contrast, two other trials^12^, ^16^ administered a tapering dose of 1, 3, and 10 μg for cat dander allergen extract and 0.1, 0.2, and 0.5 ml of 20,000 PNU/mL for grass pollen extract. Three trials^11^, ^26^, ^28^ assessed the long term outcome up to three years since the initiation of treatment. Placebo was used as a control group in 10 trials^10^, ^12^, ^13^, ^18^, ^23^, ^24^, ^25^, ^26^, ^27^, ^28^ while two trials (including a pilot study by Hylander et al.)^10^, ^11^ used a SCIT control group.

### 3.3 Outcomes

Three trials reported the symptoms score.^13^, ^25^, ^27^ Symptoms score was scored on a 0–3 points scale corresponding to no symptoms, mild, moderate or severe symptoms for two trials^13^, ^27^ while one trial^25^ scored on 0–4 points scale based on the frequency of symptoms: daily (4 points); every second day (3 points); 1–3 days per week (2 points); occasionally (1 point); never (0 points). Medication score was reported in three trials.^13^, ^25^, ^27^ The medication score was calculated based on the use of daily medications (consisting of an oral antihistamine, antihistamine eye drop, nasal corticosteroid and oral corticosteroid). The maximum daily points were 20 in one trial^27^ and 24 in another trial.^13^ Five trials^13^, ^18^, ^26^, ^27^, ^28^ reported the combined symptoms medication score. The combined symptoms medication score is the sum of the daily symptoms score and medication score. Eight trials^10^, ^11^, ^13^, ^23^, ^24^, ^25^, ^28^ reported the overall improvement score. The overall improvement score was reported in a visual analog scale ranging from zero (unchanged symptoms or no improvement) to 10 (total symptoms relief or complete recovery) when asked to compare allergic symptoms during recent pollen season versus the previous experienced season before the treatment started. Three trials^10^, ^23^, ^24^ reported the rescue medication in which they reported the number of patients with reduced rescue medication. An oral antihistamine, intranasal corticosteroid nasal spray and antihistamine eye drop were used as rescue medication in all included trials while ß_2_‐agonist and steroid inhaler were additionally given to the asthmatic patients. We analyzed the reduction of rescue medication based on the reduction of oral antihistamine and inhaled ß_2_‐agonist.

All included trials^10^, ^11^, ^12^, ^13^, ^18^, ^23^, ^24^, ^25^, ^26^, ^27^, ^28^ reported secondary outcomes. Adverse events of the treatment were reported based on the number of patients who developed symptoms after injection was given, and the severity of side effects as described. One trial^28^ reported the occurrence of adverse events based on the number of injections. The time points of measurement vary between trials: first‐hour post‐injection^13^, ^26^, ^27^; 24 h post‐injection^10^, ^23^, ^25^, ^28^; 1 week post‐injection^26^; 88 days post‐injection^12^; 4 months post‐injection^11^ and two trials^18^, ^24^ did not mention the exact time of measurement. The adverse events include local swelling at site of injection,^10^, ^12^, ^13^, ^23^, ^24^, ^25^, ^26^, ^27^ abdominal symptoms,^10^, ^11^, ^23^, ^24^ nonspecific symptoms such as headache, fatigue and muscle soreness^12^, ^13^, ^24^, ^26^ and eye and nasal itchiness.^10^, ^12^, ^13^, ^23^, ^24^, ^28^ Two trials^24^, ^26^ that reported the pre and post‐treatment serum IgE level were included for the analysis. Four trials^12^, ^13^, ^24^, ^25^ reported the quality of life after treatment. Quality of life was measured with Juniper Rhinoconjunctivitis Quality of Life Questionnaire (RQLQ)^29^ for two trials^13^, ^24^ while one trial assessed using the Juniper Asthma Quality of Life Questionnaire (AQLQ),^30^ in which the higher scores reflect the worse quality of life. Allergen sensitivity was measured by the reaction of wheal change via a skin prick test. There were two trials,^13^, ^24^ that reported mean change based on the size of wheal after skin prick tests while one trial^12^ reported post‐treatment allergen tolerance in titrated skin prick tests. Subgroup analysis was not performed due to inadequate data.

### 3.4 Risk of bias in included studies

**FIGURE 2:**
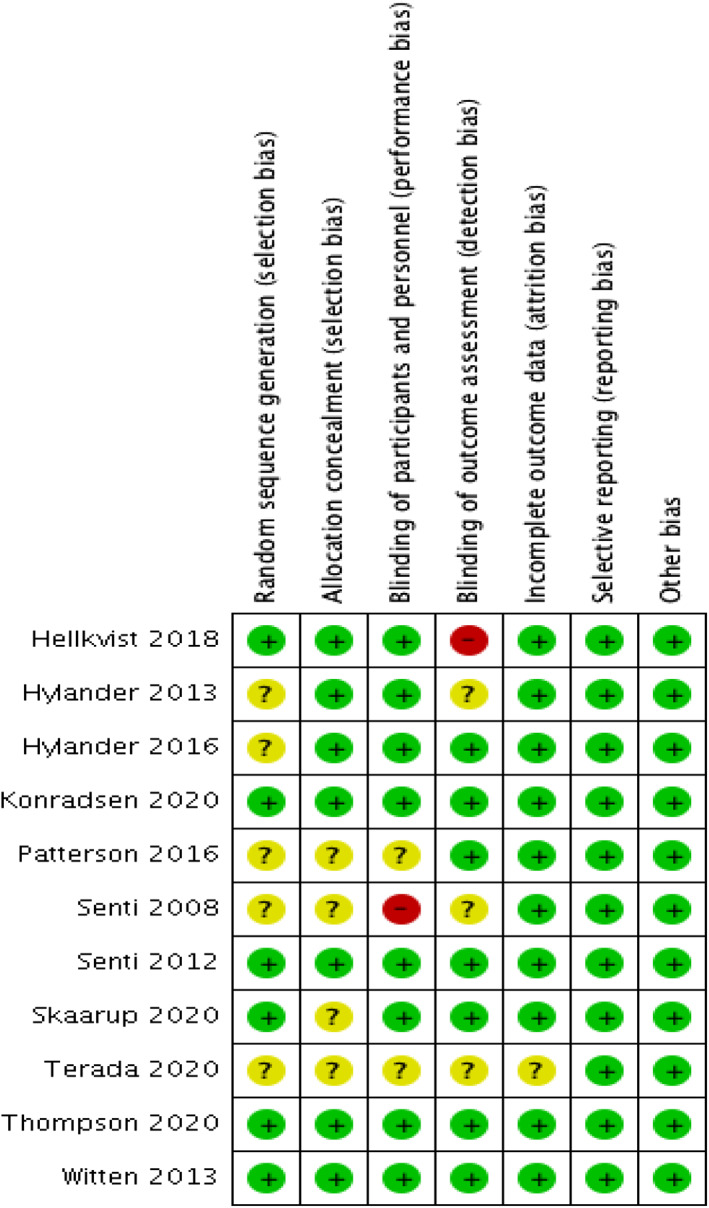
Risk of bias summary for individual study

The assessment of the risk of bias for individual trials is presented in Figure 2. Seven trials^10^, ^12^, ^23^, ^24^, ^25^, ^26^, ^27^ described the method of randomization used. Four trials^10^, ^23^, ^24^, ^25^ randomized the participants using simple randomization with a ratio of one placebo to one patient who received the intervention. Three trials^12^, ^25^, ^27^ randomized the participants according to a pre‐printed allocation list generated by a private analytical company (IKFE GmbH, Germany),^12^ randomizer.org ^26^ and REDCap Cloud EDC system^27^ using block randomization. The randomization method was not reported in five trials^10^, ^11^, ^18^, ^23^, ^28^; thus, we considered random sequence generation as an unclear risk bias. Allocation concealment was not clear in four trials.^11^, ^18^, ^26^, ^28^

### 3.5 Blinding

Placebo was used as the control group in 10 trials,^10^, ^12^, ^13^, ^18^, ^23^, ^24^, ^25^, ^26^, ^27^, ^28^ while SCIT was used as the control group by Senti et al.^11^ and in a pilot study by Hylander et al.^10^ Both patients and investigators were blinded in eight trials.^10^, ^12^, ^13^, ^23^, ^24^, ^25^, ^26^, ^27^ The control group in one trial^11^ received subcutaneous injection as immunotherapy; thus, both patient and investigator were not blinded throughout the study. Two trials^18^, ^28^ did not describe blinding. One trial^24^ had detection bias due to an error during the outcome assessment.

### 3.6 Incomplete outcome data

All trials^10^, ^11^, ^12^, ^13^, ^18^, ^23^, ^24^, ^25^, ^26^, ^27^, ^28^ were carried out as an intention‐to‐treat analysis. The non‐response rate for seven trials^11^, ^13^, ^23^, ^24^, ^25^, ^26^, ^28^ were balanced (less than 20%), and the reasons were similar between the groups. Two trials^23^, ^24^ recorded individual's withdrawal from the study after the first injection due to side‐effects at local injection site; however, they were included in the safety analysis.

### 3.7 Selective reporting

All 11 trials^10^, ^11^, ^12^, ^13^, ^18^, ^23^, ^24^, ^25^, ^26^, ^27^, ^28^ reported the outcomes as specified in their method section. All trials were prospectively registered in the WHO ICTRP and www.clinicaltrials.gov except for two trials.^10^, ^28^ The outcomes listed in the registered protocol were those reported.

### 3.8 Primary outcomes

**FIGURE 3:**
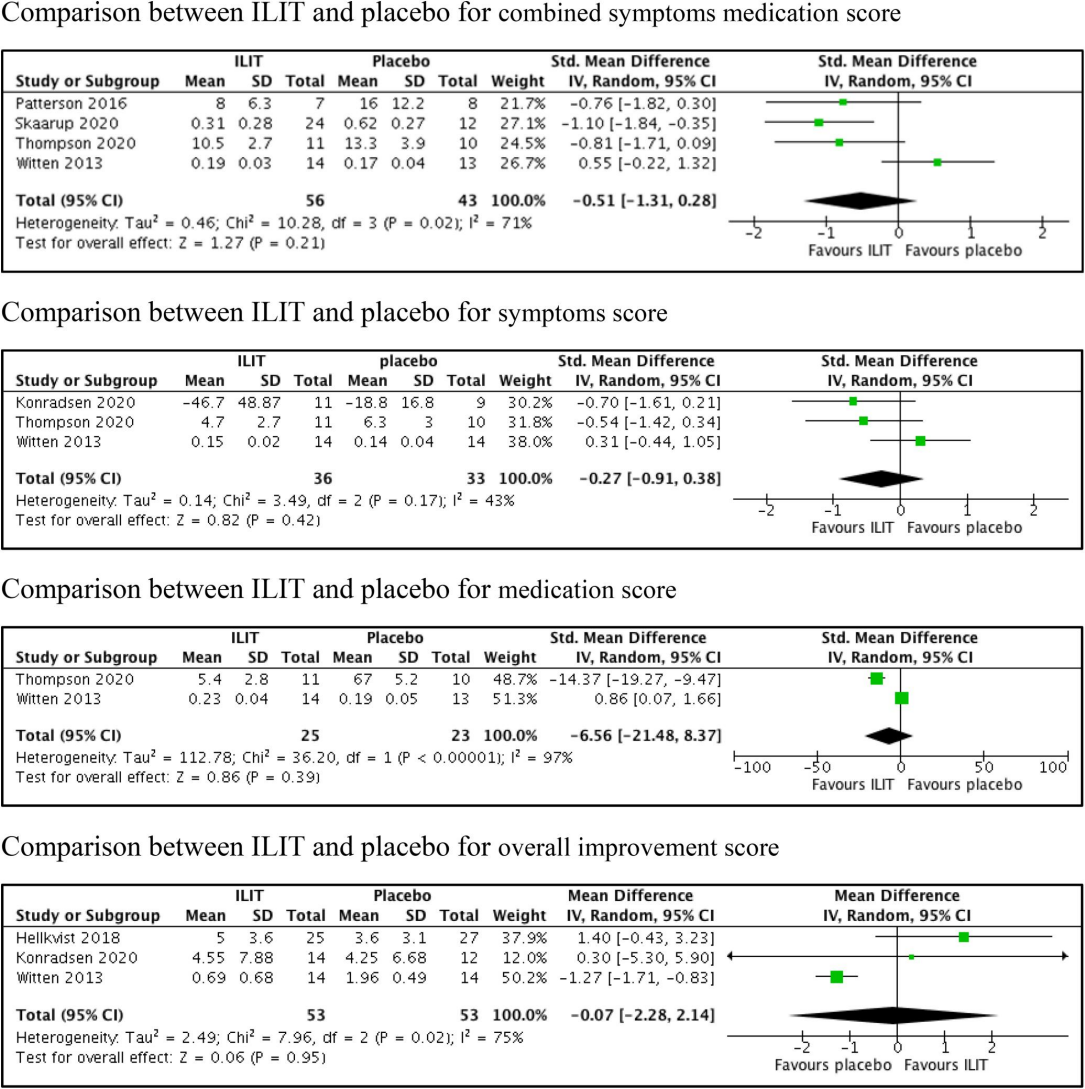
Primary outcomes of intralymphatic immunotherapy versus placebo

There are two comparisons for this review (i) ILIT versus placebo and (ii) ILIT versus SCIT. There are 10 trials^10^, ^11^, ^12^, ^13^, ^18^, ^23^, ^24^, ^25^, ^27^, ^28^ involved in the former, while two trials,^10^, ^11^ including a pilot study in the latter. The trials for comparing ILIT versus SCIT could not be analyzed as each trial assessed different outcomes. Nine out of 11 trials^10^, ^11^, ^13^, ^18^, ^23^, ^24^, ^25^, ^26^, ^27^ contributed to the primary outcomes. Primary outcomes include combined symptoms medication score, medication score, symptoms score, medication reduction and overall improvement score. All trials contributed to the secondary outcomes. Four trials^13^, ^18^, ^26^, ^27^ reported no difference in the combined symptoms and medication score following treatment between ILIT and placebo (SMD −0.51, 95% CI −1.31 to 0.28; *p* = 0.210; *I* ^2^ = 71%; 4 trials, 99 participants; low certainty evidence) (Figure 3, Table 3). Three trials^13^, ^25^, ^27^ showed no difference in symptoms score following treatment between ILIT and placebo (SMD −0.27, 95% CI −0.91 to 0.38; *p* = 0.420; *I* ^2^ = 43%; 3 trials, 69 participants; moderate certainty evidence) (Figure 3, Table 3). Two trials^13^, ^27^ described no difference in medication score following treatment between ILIT and placebo (SMD −6.56, 95% CI −21.48 to 8.37; *p* = 0.390; *I* ^2^ = 97%; 2 trials, 48 participants; very low certainty evidence) (Figure 3, Table 3). One trial^24^ reported that reduction of rescue medication was higher in ILIT compared to placebo (RR 12.32, 95% CI 0.72 to 211.79; *p* = 0.080; 1 trial, 51 participants). Three trials^13^, ^24^, ^25^ reported no difference in the overall improvement score between ILIT and placebo (MD −0.07, 95% CI −2.28 to 2.14; *p* = 0.950; *I* ^2^ = 75%; 3 trials, 106 participants; low certainty evidence) (Figure 3, Table 3).

**TABLE 3:** Summary of findings for intralymphatic immunotherapy versus placebo in treating allergic rhinitis

Intralymphatic immunotherapy compared to placebo for allergic rhinitis					
Patient or population: Allergic rhinitis Intervention: Intralymphatic immunotherapyComparison: Placebo					
Outcomes	No of participants (studies)	Certainty of the evidence (GRADE)	Relative effect (95% CI)	Anticipated absolute effects^a^ (95% CI)	
				Risk with placebo	Risk difference with allergen via ILIT
Symptom score	69 (3 RCTs)	⊕⊕⊕⊝	‐	The mean symptom score was 0	SMD 0.27 lower (0.91 lower to 0.38 higher)
		MODERATE^b^			
Local swelling	228 (8 RCTs)	⊕⊕⊝⊝	RR 4.51 (0.81 to 25.06)	Study population	
		LOW^b^ ^,^ ^c^		139 per 1000	487 more per 1000 (26 fewer to 1000 more)
Specific Ig‐ E levels	85 (2 RCTs)	⊕⊕⊝⊝	‐	The mean specific Ig‐ E levels was 0	MD 5.63 higher (0.71 higher to 10.55 higher)
		LOW^d^			
Quality of life	88 (3 RCTs)	⊕⊕⊝⊝	‐	The mean quality of life was 0	SMD 0.1 lower (0.86 lower to 0.67 higher)
		LOW^d^ ^,^ ^e^			
Combined symptoms medication score	99 (4 RCTs)	⊕⊕⊝⊝	‐	The mean combined symptoms medication score was 0	SMD 0.51 lower (1.31 lower to 0.28 higher)
		LOW^b^ ^,^ ^c^			
Medication score	48 (2 RCTs)	⊕⊝⊝⊝	‐	The mean medication score was 0	SMD 6.56 lower (21.48 lower to 8.37 higher)
		VERY LOW^c^ ^,^ ^d^			
Overall improvement score	106 (3 RCTs)	⊕⊕⊝⊝	‐	The mean overall improvement score was 0	MD 0.07 lower (2.28 lower to 2.14 higher)
		LOW^c^ ^,^ ^d^			

Abbreviations: CI, Confidence interval; RR, Risk ratio; RCTs: randomized controlled trials; ILIT, Intralymphatic immunotherapy.

^a^ **The risk in the intervention group** (and its 95% confidence interval) is based on the assumed risk in the comparison group and the **relative effect** of the intervention (and its 95% CI).

^b^ Small number of participants (<400).

^c^ substantial heterogeneity.

^d^ Very small number participants (<100).

^e^ Moderate heterogeneity with wide confidence interval.

For ILIT versus SCIT group, one trial^10^ showed no difference in symptoms improvement during the next pollen season after the treatment (MD −0.60, 95% CI −1.74 to 0.54; *p* = 0.300; 1 trial; 13 participants). One trial^11^ comparing ILIT and SCIT, reported the reduction of rescue medication was higher in SCIT group compared to ILIT (RR 0.64, 95% CI 0.45 to 0.93; *p* = 0.020; 1 trial, 107 participants).

### 3.9 Secondary outcomes

**FIGURE 4:**
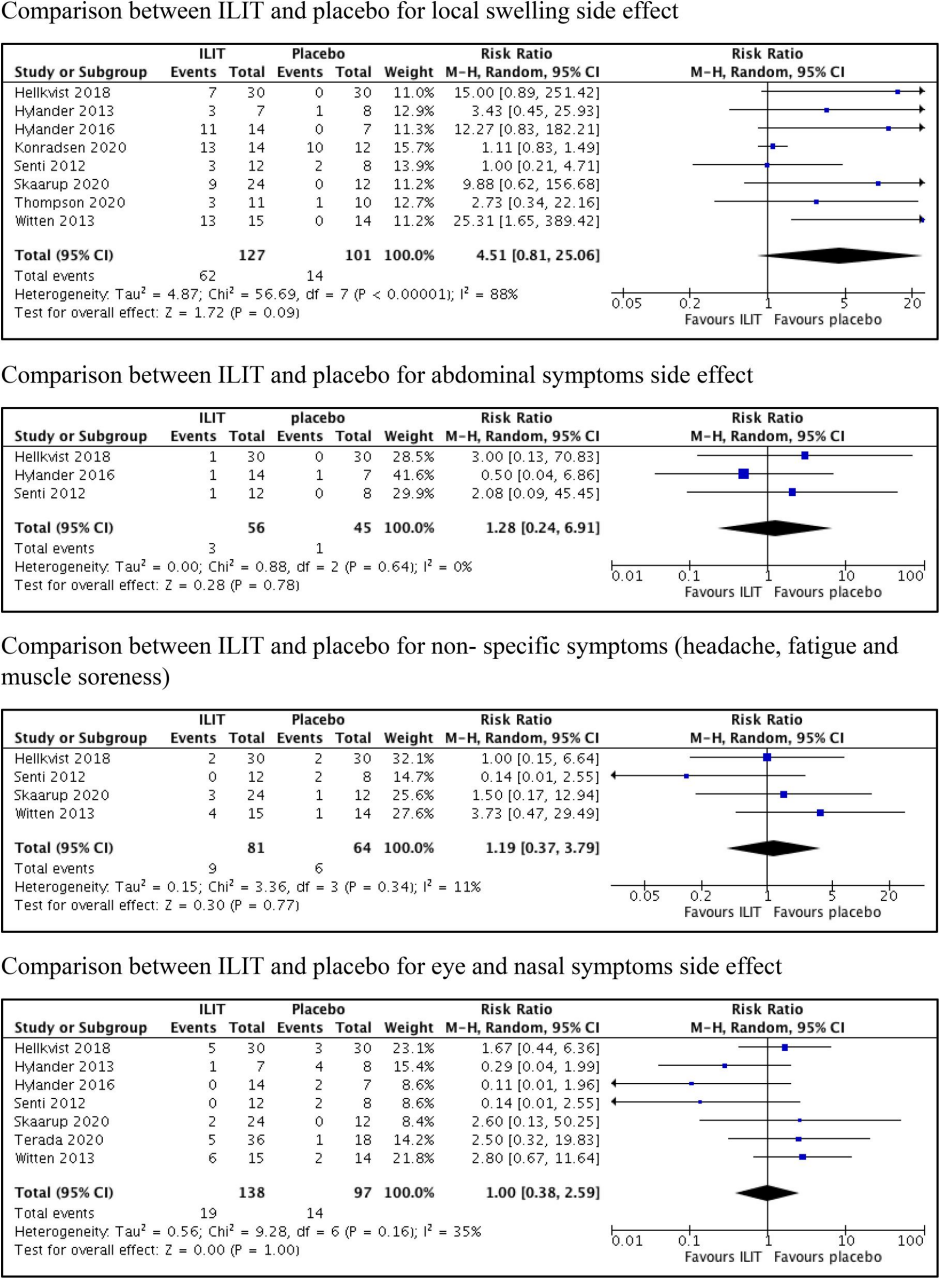
Secondary outcomes (adverse events) of intralymphatic immunotherapy versus placebo

Intralymphatic immunotherapy showed no difference for local swelling at the site of injection in eight trials^10^, ^12^, ^13^, ^23^, ^24^, ^25^, ^26^, ^27^ compared to placebo (RR 4.51, 95% CI 0.81 to 25.06; *p* = 0.090; *I* ^2^ = 88%; 8 trials, 228 participants; low certainty evidence) (Figure 4, Table 3). Likewise, no difference was documented for other adverse events (local and systemic adverse effects) between ILIT and placebo; abdominal symptoms^12^, ^23^, ^24^ (RR 1.28, 95% CI 0.24 to 6.91; *p* = 0.780; *I* ^2^ = 0%; 3 trials, 101 participants; moderate certainty evidence) (Figure 4), non‐specific symptoms such as fatigue, headache and muscle soreness^12^, ^13^, ^24^, ^26^ (RR 1.19, 95% CI 0.37 to 3.79; *p* = 0.770; *I* ^2^ = 11%; 4 trials, 145 participants; moderate certainty evidence) (Figure 4) and eye and nasal symptoms^10^, ^12^, ^13^, ^23^, ^24^, ^26^, ^28^ (RR 1.00, 95% CI 0.38 to 2.59; *p* = 1.000; *I* ^2^ = 35%; 7 trials, 235 participants; low certainty evidence) (Figure 4). In the ILIT versus SCIT group, one trial^11^ compared cutaneous reaction post injection for ILIT and SCIT. SCIT showed three times more incidence of cutaneous reactions compared to ILIT (RR 0.31, 95% CI 0.13 to 0.72; *p* = 0.007; 1 trial, 114 participants). One trial^11^ comparing ILIT and SCIT demonstrated no difference for the incidence of dyspnea following treatment (RR 0.19, 95% CI 0.01 to 3.80; *p* = 0.270; 1 trial, 114 participants).

**FIGURE 5:**
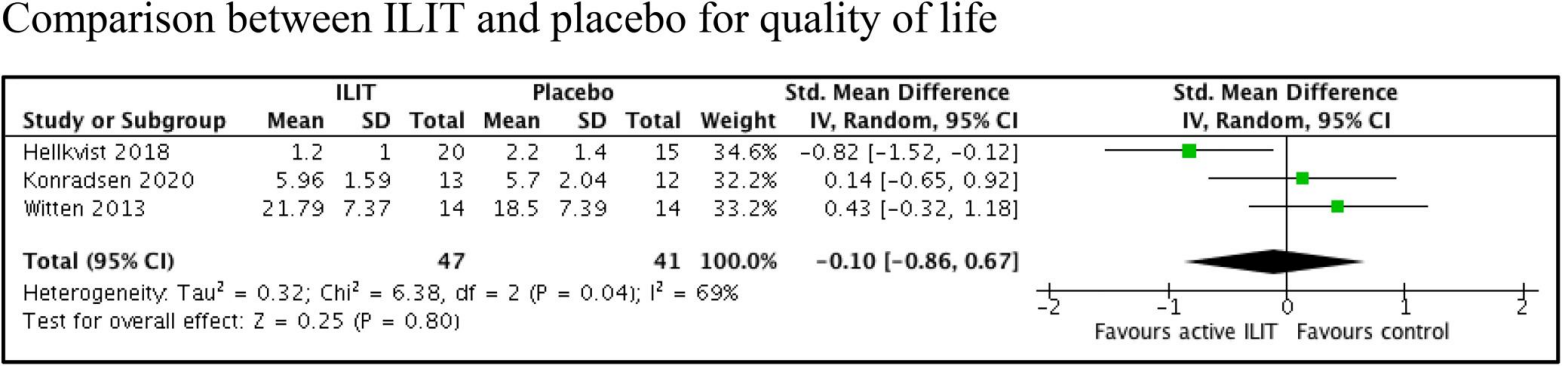
Secondary outcomes (quality of life) of intralymphatic immunotherapy versus placebo

Intralymphatic immunotherapy showed increase in specific IgE level following treatment compared to placebo in two trials^24^, ^26^ (MD 5.63, 95% CI 0.71 to 10.55; *p* = 0.020; *I* ^2^ = 0%; 2 trials, 85 participants; low certainty evidence) (Table 3). One trial^10^ reported ILIT showed a faster reduction of serum specific IgE level within three months after the completion of treatment as compared to three years for SCIT (MD −14.73, 95% CI −24.77 to 4.69; *p* = 0.004; 1 trial, 13 participants). Intralymphatic immunotherapy showed reduced reaction of skin prick test by 0.88 mm compared to placebo in two trials^13^, ^24^ (MD −0.88, 95% CI −1.53 to −0.23; *p* = 0.008; 1 trial, 27 participants). Three trials^13^, ^24^, ^25^ reported no difference in the quality of life between ILIT and placebo (SMD −0.10, 95% CI −0.86 to 0.67; *p* = 0.800; *I* ^2^ = 69%; 3 trials, 88 participants; low certainty evidence) (Figure 5, Table 3).

## 4 DISCUSSION

This review found the efficacy and safety of ILIT are not different from placebo in AR management. Most results showed no difference between ILIT and control for the primary and secondary outcomes. Despite most trials reported swelling at the injection site as being the most common adverse event in their patients, the present review found no significant difference in the overall adverse events between ILIT and placebo. Whilst most trials did not report severe reaction, a trial by Lee et al.^20^ reported two patients experienced anaphylaxis following ILIT. Remarkably, the events occurred after the first injection in one patient and second injection in another patient. Both events were attributed to the use of non‐standardized allergen extract as compared to standard allergen extract used in other trials. Albeit the limited availability of trials comparing ILIT with other AIT, the results of two trials^10^, ^11^ suggest that ILIT has the advantage of less cutaneous reaction and dyspnea to SCIT in addition to the benefit of faster reduction of serum specific IgE level.

We noted the inconsistency of the administration of ILIT in a 2‐week interval in one trial^13^ compared with a 4‐week interval for others which can be considered as an outlier. The general recommendation for ILIT is three doses given in a 4‐week interval for the development of successive waves of antigen‐specific immune responses^25^ but different doses and interval between injection were used among the trials. Although, the impact of this incongruity on the outcome is arguable, standardization of the injection interval is required to ensure comparable outcomes. If further trials are to be conducted to analyze ILIT, using a standardized interval and number of doses to administer ILIT coupled with a suitable outcome measurement such as combined symptoms and medication score would represent a better reflection of the actual potential of ILIT. In trials involving AIT, symptom and medication scores are often assessed independently. The World Allergy Organization taskforce^31^ recommended the standardization of primary endpoint in AIT trial by using the combined symptoms and medication score. As the severity of symptoms entails a higher frequency of medication consumption, this link will be better replicated by combining the symptoms and medication score to indicate their equivalent contribution.

Interestingly, the results of our review contradict the findings of a meta‐analysis recently published by Hoang et al.^32^ which found ILIT confers short term improvement of combined symptoms medication score. The discrepancy can be explained by the different statistical models applied for the meta‐analysis, the fixed‐effect model in their review and the random‐effects model in the present review. We used the random‐effects model as it was advocated to account for the heterogeneity across the studies.^16^ For the analysis results to be generalized to a population, it should consider the varying resources available to the investigators from study to study which makes it unlikely that the intervention effects across the studies to be identical.

### 4.1 Limitations

High heterogeneity among the trials could be contributed by different allergens exhibiting different immunogenicity effect. One trial^24^ treated AR patients with polysensitization using grass and birch allergen demonstrated an opposite effect on serological IgE levels. While grass‐specific IgE showed a transient increase, birch‐specific IgE did not exhibit any alteration. The transient increase of IgE level is attributed to an early phase of desensitization, similarly seen during conventional AIT. Hence, the different immunogenicity effect of different allergen used for ILIT needs to be further investigated.

## 5 CONCLUSIONS

Intralymphatic immunotherapy possibly has a role in the treatment of AR patients. This review found it is safe but not effective, which could be contributed by the high variation amongst the trials. Future trials should involve larger numbers of participants and report standardized administration of ILIT and outcome measures.

## CONFLICT OF INTERESTS

Sarah K. Wise is a consultant for NeurENT, Stryker and advisory board member for OptiNose, SinopSys Surgical, ALK‐Abello, Genentech. The authors have no other funding, financial relationships, or conflicts of interest to disclose.

## AUTHOR CONTRIBUTIONS

Nor Rahimah Aini, Norhayati Mohd Noor, Mohd Khairi Md Daud, Sarah K. Wise, and Baharudin Abdullah conceptualized and performed the study. Nor Rahimah Aini and Norhayati Mohd Noor made the statistical analysis. Nor Rahimah Aini, Norhayati Mohd Noor, Mohd Khairi Md Daud, Sarah K. Wise, and Baharudin Abdullah wrote the manuscript. All authors approved the final version of the manuscript before submission.

## Supporting information

Supplementary Material
