# Supplementary material for: Efficacy and safety of intralymphatic immunotherapy in allergic rhinitis: A systematic review and meta‐analysis
Source: Clin Transl Allergy. 2021 Aug 17;11(6):e12055. doi: 10.1002/clt2.12055 (PMC8369948; doi:10.1002/clt2.12055)
Supplement: Supplementary file 1 — Supplementary Material [file CLT2-11-e12055-s001.docx]

**Matrix-based Method for Solving Decision Domains of Neighbourhood Multigranulation Decision-theoretic Rough S**ets

Jiajun Chen^1*^, Shuhao Yu^1^, Wenjie Wei^2^ and Yan Ma^1^

^1^College of Electronics and Information Engineering, West Anhui University, Lu’an, China.

^2^College of Electronics and Information Engineering, Tongji University, Shanghai, China

*corresponding.[chenjj@wxc.edu.cn](mailto:chenjj@wxc.edu.cn)

Abstract: It is more and more important to analyze and process the complex data for gaining more valuable knowledge and making more accurate decisions. Multigranulation decision theory based on conditional probability and cost loss has the advantage of processing decision-making problems from multi-level and multi-angle, and the neighbourhood rough set model (NRS) can facilitate the analysis and processing of numerical or mixed type data, and can address the limitation of multigranulation decision-theoretic rough sets (MG-DTRS) which is not easy to cope with complex data. Based on in-depth study of hybrid-valued decision systems and MG-DTRS models, this paper analyzed neighbourhood MG-DTRS (NMG-DTRS) deeply by fusing MG-DTRS and NRS, a matrix-based approach for approximation sets of NMG-DTRS model was proposed on the basis of the matrix representations of concepts, the positive, boundary and negative domains were constructed from the matrix perspective, and the concept of positive decision recognition rate was introduced. Furthermore, we explored the related properties of NMG-DTRS model, and designed and described the corresponding solving algorithms in detail. Finally, some experimental results were employed not only verified the effectiveness and feasibility of the proposed algorithm, but also showed the relationship between the decision recognition rate and the granularity and threshold.

1. Introduction

Rough set theory^[1-2]^ is an important theory in the fields of artificial intelligence, it is a new mathematical tool to cope with uncertain and imprecise data, which was proposed by Pawlak in 1982, and has extensive applications in various fields, such as government decision-making, financial analysis, data mining and medical system. Decision-theoretic rough sets model(DTRS)^[3-4]^ is an important decision-making model based on probability and risk-cost sensitivity for solving practical decision problems with uncertain data, and multigranulation decision theory is devoted to analyze target decision objects from multi-level and multi-angle^[5-8]^. DTRS and a large number of extended models have been studied to address the corresponding requirements in recent years^[9-13]^. However, most proposed analysis methods in the light of the classical rough set theory can only be used to process single type of data, such as symbolic data, and there are some limitations in the processing of complex data such as numerical or hybrid-valued type decision systems^[14]^, furthermore, with the increasing application of data science and artificial intelligence technology, data from all walks of life are becoming more huge, complex, diverse and uncertain, and people need more comprehensive and diversified analysis and processing of these complex data in order to obtain more valuable knowledge and make more accurate decisions, so the research of multi-granulation decision making methods for numerical data and mixed data has been paid more and more important. Qian et al.^[15]^ developed firstly MG-DTRS model by the combination of multigranulation rough sets and Bayesian decision theory, however, the application scope of the models in the light of the equivalence relation was limited because it was not easy to cope with all kinds of complex data, especially hybrid-valued type information systems with numerical type and categorical type properties. In order to address the limitation, Lin et al. put forward the neighbourhood rough set models (NRS) in literature [16-17], in the following years, several types of generalized NRS models were widely used in various domains. Literature [18] discussed DTRS model in the neighborhood system environment, literature [14] deeply analyzed neighborhood DTRS(NDTRS) and MG-DTRS model, and proposed an incomplete neighborhood multigranulation decision-theoretic rough set (NMG-DTRS) model, reference [19] investigated neighborhood multigranulation rough sets (NMRS) and attribute reduction method for incomplete information systems with symbolic type and numerical type properties. In addition to the mentioned frameworks, some important insights based on these models have been explored, such as the application of matrix technology. In rough set theory, matrix has the advantages of intuitive and simple knowledge representation and reasoning, so, matrix-based methods has been widely used in some fields of rough set^[20-28]^, including decision making information systems^[22,28]^, covering approximate spaces^[26-28]^, neighbourhood information systems^[20,23,25]^ and multigranulation spaces^[23,25,28]^. Literature[23] discussed matrix-based approaches for dynamic updating approximations in multigranulation rough sets, literature[20] and literature[25] studied respectively the problems of decision domains updating and approximations updating in neighborhood multigranulation space by introducing the matrix technique into neighborhood multigranulation rough set, and the description of maximum and minimum covering rough sets was discussed in detail based on matrix technology in literature[26]. Among the above achievements, there were few researches on NMG-DTRS in complex information system, although literature[22] illustrated the matrix approach for decision-theoretic rough sets, but it is not suitable for neighborhood information systems in multi-granularity environment. Literature[14] explored two types of NMG-DTRS in detail based on incomplete hybrid -valued decision system, however, the knowledge representation based on the models are difficult to understand and has some computational complexity. In this paper, in view of the characteristics of matrix in rough set context, we introduced the matrix technique into NMG-DTRS and proposed a matrix-based method for solving decision domains of NMG-DTRS model for hybrid-valued decision information systems, the positive, boundary and negative domains were constructed from the matrix perspective. Furthermore, we explored the related properties of NMG-DTRS model, and the corresponding solving algorithms based on the proposed method were designed and described in detail

The other sections of the paper were organized as follows. First, the fundamental knowledge of DTRS, MG-DTRS and NMG-DTRS was reviewed briefly in Section 2, in Section3, a novel matrix-based method for approximation sets of NMG-DTRS model was proposed on the basis of the matrix representations of a series of concepts, and the positive, boundary and negative domains were constructed

based on NMG-DTRS model. Furthermore, the related properties of NMG-DTRS model and implementation algorithms were discussed in depth. Finally, some experimental results were employed to prove the algorithm is feasible and effective.

1. ****Preliminary knowledge****

Here we reviewed some related works of DTRS, MG-DTRS and neighborhood MG-DTRS models in this section.

- 1. DTRS

$IS=(U,A=C\cup D,V,f)$ is a given decision table information system and where U represents the target space object set, $C$ and $D$ ($C\cap D=\emptyset)$are condition attributes and decision attributes respectively,$V=\bigcup_{c\in A} V_{c}$,$V_{c}$ indicates a nonempty value set of$c\in A$, and the mapping function f is expressed by $f:U\times A\to V$. For any given nonempty subset$P\subseteq C$, an object $x$ can been represented by its equivalence relations${[x]}_{P}=\{y\in U|\forall c\in P(f_{c}\left( x \right)=f_{c}\left( y \right))\}$. For any $X'\subseteq U$, we can obtain the lower approximation sets and the upper approximation sets of $X'$ on subset $P$ by $\underline{\mathrm{apr}_{P}}\left( X' \right)=\left\{ x\in U | \left[ x \right]_{P}\subseteq X' \right\}$and$\overline{\mathrm{apr}_{P}}\left( X' \right)=\left\{ x\in U | \left[ x \right]_{P}\cap X'\neq\emptyset\right\}.$In the light of the above description, then $\mathrm{POS}_{P}\left( X' \right)=\underline{\mathrm{apr}_{P}}\left( X' \right),\mathrm{BND}_{P}\left( X' \right)=\overline{\mathrm{apr}_{P}}\left( X' \right)-\underline{\mathrm{apr}_{P}}\left( X' \right)$ and$\mathrm{NEG}_{P}\left( X' \right)=U-\overline{\mathrm{apr}_{P}}\left( X' \right)$are called the positive domains, boundary domains and negative domains of $X'$ about the subset $P$ respectively.

In DTRS model, suppose $\delta=\{X,X^{\mathcal{\mathcal{L}}}\}$ indicates the state set for any object $x$, namely object $x$ belongs to category $X$ and belong to category $X^{\mathcal{\mathcal{L}}}$. Then the probability of object $x$ belongs to $X$ can be obtained by $P_{r}\left( X | \left[ x \right] \right)=\frac{|X\cap[x]|}{|\left[ x \right]|})$, and the probability of $x$ not in $X$ is $P_{r}\left( X^{\mathcal{\mathcal{L}}} | \left[ x \right] \right)=1-p\left( X | \left[ x \right] \right)$. For $\forall X\subseteq U$, order $\mathrm{POS}_{\left( \alpha,\beta\right)}\left( X \right)$, $\mathrm{BND}_{\left( \alpha,\beta\right)}\left( X \right)$ and $\mathrm{NEG}_{\left( \alpha,\beta\right)}\left( X \right)$indicate positive domains, boundary domains and negative domains of X based on thresholds$(\alpha,\beta)$. In view of Bayesian decision procedure, let $\lambda_{\mathrm{PP}}$,$\lambda_{\mathrm{BP}}$ and $\lambda_{\mathrm{NP}}$ represent the loss functions of classifying object $x$ into domains $\mathrm{POS}_{\left( \alpha,\beta\right)}\left( X \right)$,$\mathrm{BND}_{\left( \alpha,\beta\right)}\left( X \right)$ and $\mathrm{NEG}_{\left( \alpha,\beta\right)}\left( X \right)$ respectively when object $x$belongs to category $X$; $\lambda_{\mathrm{PN}}, \lambda_{\mathrm{BN}}$ and $\lambda_{\mathrm{NN}}$ represent the loss functions that it be classified in domains$\mathrm{POS}_{\left( \alpha,\beta\right)}\left( X \right)$, $\mathrm{BND}_{\left( \alpha,\beta\right)}\left( X \right)$ and $\mathrm{NEG}_{\left( \alpha,\beta\right)}\left( X \right)$ when object $x$ is not in category $X$ respectively. Consider the reasonable assumption that the losses of taking right action is less than or equal to the losses of taking wrong action, namely, we can know $\lambda_{\mathrm{PP}}\leq\lambda_{\mathrm{BP}}<\lambda_{\mathrm{NP}}$ and $\lambda_{\mathrm{NN}}\leq\lambda_{\mathrm{BN}}<\lambda_{\mathrm{PN}}$, the thresholds$(\alpha,\beta)$ can be obtained from all loss functions by formula (1). The detailed derivation process is shown in reference[4], where$1\geq\alpha>\beta\geq0$.


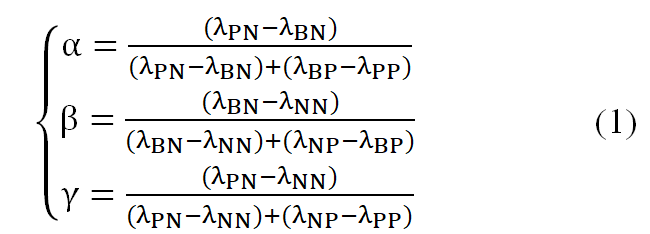
Based on Bayesian decision procedure, for $\forall X'\subseteq U, P\subseteq C$, the decision rules closely related to the probability of object $x$ based on thresholds$(\alpha,\beta)$ in DTRS model can been obtained:

$$\left( 1 \right)\mathrm{if}\mathrm{probability}P_{r}\left( X' | \left[ x \right]_{P} \right)\geq\alpha is satisfied, then$$

$$\mathrm{object}x\in{POS}_{\left( \alpha,\beta\right)}(X')$$

$$\left( 2 \right)\mathrm{if}\mathrm{probability}\beta\leq P_{r}\left( X' | \left[ x \right]_{P} \right) <\alpha is satisfied ,then$$

$$\mathrm{object}x\in{BND}_{\left( \alpha,\beta\right)}(X')$$

$$\left( 3 \right)\mathrm{if}\mathrm{probability}P_{r}\left( X' | \left[ x \right]_{P} \right)<\beta is satisfied ,then$$

$$\mathrm{object}x\in{NEG}_{\left( \alpha,\beta\right)}(X')$$

For the given decision information system$IS=(U,A=C\cup D,V,f)$, suppose $\pi_{D}=\{D_{1},D_{2},\cdots,D_{m}\}$ indicates the partitions on the target space $U$ with respect to decision attribute $D\in A$, for any attribute subset $B\subseteq C$ and $\forall x\in U$, then we can get the probabilistic lower and upper approximations of$\pi_{D}$ about B in DTRS model as follows: $\underline{D_{B}^{\left( \alpha,\beta\right)}(X')}=\{\cup x|P_{r}\left( \pi_{D} | \left[ x \right]_{B} \right)\geq\alpha,x\in U\}$ (2)

$\overline{D_{B}^{\left( \alpha,\beta\right)}(X')}==\{\cup x|P_{r}\left( \pi_{D} | \left[ x \right]_{B} \right)\geq\alpha,x\in U\}$ (3)

- 1. MG-DTRS

MG-DTRS is a rough decision theory in the context of multigranulation, which combining DTRS theory and the multigranulation idea, and can been widely used in many fields, including multi-source data analysis, distributive information systems and intelligent decision making from data with multi dimensions. In this section, the pessimistic MG-DTRS model and the optimistic one were discussed as two focuses in multigranulation rough sets. The relevant definitions were described as follows.

Definition 1. Suppose $IS=(U,A=C\cup D,V,f)$be a decision table information system, and where the universe $U=\{x_{i}|i=1,2,\cdots,n\}$, $A_{1},A_{2},\cdots A_{m}\subseteq A$ are m granular structures, $\left[ x_{i} \right]_{A_{k}}$denotes equivalence granule of $x_{i}$ about the *k*th granular structure $A_{k}(k=1,2,\cdots,m)$. For any $X'\subseteq U$, then the approximations of $X'$ in the optimistic MG-DTRS model are expressed by:

$\underline{\sum_{k=1}^{m} A_{k}^{O,\alpha}\left( X' \right)}= \{x_{i}\in U:\bigvee_{k=1}^{m} P_{r}\left( X' | \left[ x_{i} \right]_{A_{k}} \right)\geq\alpha$}

$\overline{\sum_{k=1}^{m} A_{k}^{O,\beta}\left( X' \right)}=\{x_{i}\in U:\bigwedge_{k=1}^{m} P_{r}\left( X' | \left[ x_{i} \right]_{A_{k}} \right)\geq\beta$}

Where $\wedge$and $\vee$denotes the logic conjunction(AND) and the logic disjunction(OR) operations respectively.

**Definition 2.** Suppose $IS=(U,A=C\cup D,V,f)$be a decision table information system, and where the universe $U=\{x_{i}|i=1,2,\cdots,n\}$, $A_{1},A_{2},\cdots A_{m}\subseteq A$ are m granular structures, $\left[ x_{i} \right]_{A_{k}}$denotes equivalence granule of $x_{i}$ about the *k*th granular structure $A_{k}(k=1,2,\cdots,m)$. For any $X'\subseteq U$, then the approximations of $X'$ in the pessimistic MG-DTRS model are expressed by:

$\underline{\sum_{k=1}^{m} A_{k}^{P,\alpha}\left( X' \right)}= \{x_{i}\in U:\bigwedge_{k=1}^{m} P_{r}\left( X' | \left[ x_{i} \right]_{A_{k}} \right)\geq\alpha$}

$\overline{\sum_{k=1}^{m} A_{k}^{P,\beta}\left( X' \right)}=\{x_{i}\in U:\bigvee_{k=1}^{m} P_{r}\left( X' | \left[ x_{i} \right]_{A_{k}} \right)\geq\beta$}

Where $\wedge$and $\vee$denotes the logic conjunction(AND) and the logic disjunction(OR) operations respectively.

By **definition 1, definition 2** and the decision rules in decision-theoretic rough set model, the positive domains, boundary domains and negative domains of the optimistic MG-DTRS model can be formalized as follows, respectively.

$$\sum_{k=1}^{m} A_{k}^{OPOS,\alpha}\left( X \right)=\underline{\sum_{k=1}^{m} A_{k}^{O,\alpha}\left( X \right)}$$

$$\sum_{k=1}^{m} A_{k}^{OBND,\alpha\beta}\left( X \right)=\overline{\sum_{k=1}^{m} A_{k}^{P,\beta}\left( X \right)}-\underline{\sum_{k=1}^{m} A_{k}^{O,\alpha}\left( X \right)}$$

$$\sum_{k=1}^{m} A_{k}^{ONEG,\beta}\left( X \right)=U-\overline{\sum_{k=1}^{m} A_{k}^{P,\beta}\left( X \right)}$$

The positive domains, boundary domains and negative domains of the pessimistic MG-DTRS model can be formalized as follows respectively.

$$\sum_{k=1}^{m} A_{k}^{PPOS,\alpha}\left( X \right)=\underline{\sum_{k=1}^{m} A_{k}^{P,\alpha}\left( X \right)}$$

$$\sum_{k=1}^{m} A_{k}^{PBND,\alpha\beta}\left( X \right)=\overline{\sum_{k=1}^{m} A_{k}^{P,\beta}\left( X \right)}-\underline{\sum_{k=1}^{m} A_{k}^{P,\alpha}\left( X \right)}$$

$$\sum_{k=1}^{m} A_{k}^{PNEG,\beta}\left( X \right)=U-\overline{\sum_{k=1}^{m} A_{k}^{P,\beta}\left( X \right)}$$

- 1. NMG-DTRS

MG-DTRS models are based on classical DTRS, which uses equivalence relations to partition the target object universe and generate multiple equivalence classes as basic concepts^[21]^. However, the models based on the equivalence relation are not easy to cope with all kinds of complex data, especially hybrid-valued type information system with numerical type and categorical type properties ^[14,20]^. Therefore, neighborhood rough set (NRS) was put forward because of the advantage of facilitating the analysis and processing of numerical or mixed type data. NRS model uses neighborhood relation to partition the decision object target spaces and replaces the equivalence classes with neighborhood particles. In NMG-DTRS models, the equivalence classes based on conditional probability of classical MG-DTRS are replaced by the neighbourhood granularity, the details are as follows.

A given neighborhood information system $NIS=(U,A=C\cup D,V,f)$, where U represents the target space objects, $C$ and $D$ ($C\cap D=\emptyset)$are condition attributes and decision attributes respectively, suppose any $B\subseteq C$ be a condition attribute subset, for any object $x_{i}\in U$, then the neighborhood granularity $N_{B}\left( x_{i} \right)$of the object $x_{i}$ based on attribute subset B is defined by:$N_{B}\left( x_{i} \right)=\left\{ x_{j} \right|\Delta_{B}\left( x_{j},x_{i} \right)\leq\delta,x_{j}\in U\}$.$\delta(\delta\geq0)$ is the neighborhood radius. Where $\Delta$is a distance function, here only take Euclidean distance as a metric function and is defined as:

$\Delta_{B}\left( x_{j},x_{i} \right)=\sqrt{\sum_{k=1}^{|B|} {(b_{k}\left( x_{i} \right)-b_{k}\left( x_{j} \right))}^{2}}$. (4)

According to **Definition 1** and **Definition 2**, we can obtain the approximations in the NMG-DTRS model by substituting neighbourhood granularity for conditional equivalence classes.

**Definition 3.** Suppose $NIS=(U,A=C\cup D,V,f)$ be a neighbourhood decision table information system, where U represents the target space objects,$U=\{x_{i}|i=1,2,\cdots,n\}$ and $A_{1},A_{2},\cdots A_{m}\subseteq A$ are m granular structures, $N_{A_{k}}\left( x_{i} \right)$ denotes neighbourhood granularity of the object $x_{i}$ about granular structure $A_{k}$, where $(k=1,2,\cdots,m)$. Given the thresholds$(\alpha,\beta)$, for $\forall X\subseteq U$, then the approximation sets of $X$ based on the optimistic NMG-DTRS model are defined by:

$\underline{\sum_{k=1}^{m} N_{A_{k}}^{O,\alpha}\left( X \right)}= \{x_{i}\in U:\bigvee_{k=1}^{m} P_{r}\left( X | N_{A_{k}}\left( x_{i} \right) \right)\geq\alpha$}

$\overline{\sum_{k=1}^{m} N_{A_{k}}^{O,\beta}\left( X \right)}=\{x_{i}\in U:\bigwedge_{k=1}^{m} P_{r}\left( X | N_{A_{k}}\left( x_{i} \right) \right)\geq\beta$}

**Definition 4.** Suppose $NIS=(U,A=C\cup D,V,f)$ be a neighbourhood decision table information system, where U represents the target space objects,$U=\{x_{i}|i=1,2,\cdots,n\}$ and $A_{1},A_{2},\cdots A_{m}\subseteq A$ are m granular structures, $N_{A_{k}}\left( x_{i} \right)$ denotes neighbourhood granularity of the object $x_{i}$ about granular structure $A_{k}$, where $(k=1,2,\cdots,m)$. Given the thresholds$(\alpha,\beta)$, for $\forall X\subseteq U$, then the approximation sets of $X$ based on the pessimistic NMG-DTRS model are defined by:

$\underline{\sum_{k=1}^{m} N_{A_{k}}^{P,\alpha}\left( X \right)}= \{x_{i}\in U:\bigwedge_{k=1}^{m} P_{r}\left( X | N_{A_{k}}\left( x_{i} \right) \right)\geq\alpha$}

$\overline{\sum_{k=1}^{m} N_{A_{k}}^{P,\beta}\left( X \right)}=\{x_{i}\in U:\bigvee_{k=1}^{m} P_{r}\left( X | N_{A_{k}}\left( x_{i} \right) \right)\geq\beta$}

By **definition 3, definition 4** and the decision rules in decision-theoretic rough set model, the positive domains, negative domains and boundary domains based on the optimistic NMG-DTRS model can be formalized as follows, respectively.

$N_{\sum_{k=1}^{m} A_{k}}^{OPOS,\alpha}\left( X \right)=\underline{\sum_{k=1}^{m} N_{A_{k}}^{O,\alpha}\left( X \right)}$ (5)

$N_{\sum_{k=1}^{m} A_{k}}^{OBND,\alpha\beta}\left( X \right)=\overline{\sum_{k=1}^{m} N_{A_{k}}^{O,\beta}\left( X \right)}-\underline{\sum_{k=1}^{m} N_{A_{k}}^{O,\alpha}\left( X \right)}$ (6)

$N_{\sum_{k=1}^{m} A_{k}}^{ONEG,\beta}\left( X \right)=U-\overline{\sum_{k=1}^{m} N_{A_{k}}^{O,\beta}\left( X \right)}$ (7)

The positive domains, negative domains and boundary domains based on the pessimistic NMG-DTRS model can be formalized as follows, respectively.

$N_{\sum_{k=1}^{m} A_{k}}^{PPOS,\alpha}\left( X \right)=\underline{\sum_{k=1}^{m} N_{A_{k}}^{P,\alpha}\left( X \right)}$ (8)

$N_{\sum_{k=1}^{m} A_{k}}^{PBND,\alpha\beta}\left( X \right)=\overline{\sum_{k=1}^{m} N_{A_{k}}^{P,\beta}\left( X \right)}-\underline{\sum_{k=1}^{m} N_{A_{k}}^{P,\alpha}\left( X \right)}$ (9)

$N_{\sum_{k=1}^{m} A_{k}}^{PNEG,\beta}\left( X \right)=U-\overline{\sum_{k=1}^{m} N_{A_{k}}^{P,\beta}\left( X \right)}$ (10)

**Definition 5.** Suppose $NIS=(U,A=C\cup D,V,f)$ be a neighbourhood decision table information system, where U represents the target space objects,$U=\{x_{i}|i=1,2,\cdots,n\}$ and$A_{1},A_{2},\cdots A_{m}\subseteq A$ are m granular structures. Order $\pi_{D}=\{D_{1},D_{2},\cdots,D_{m}\}$ indicates the partitions on the target space objects $U$ with respect to decision space set$D\in A$, for $\forall D_{i}\subseteq\pi_{D}$ and thresholds$(\alpha,\beta)$, order$N_{\sum_{k=1}^{m} A_{k}}^{PPOS,\alpha}\left( D_{i} \right)$and $N_{\sum_{k=1}^{m} A_{k}}^{OPOS,\alpha}\left( D_{i} \right)$ denote the positive decision domains of $D_{i}$ in the pessimistic and optimistic NMG-DTRS model, then we have the the positive decision domains with respect to$\pi_{D}$ in the pessimistic and optimistic NMG-DTRS model as follows:

${DN}_{\sum_{k=1}^{m} A_{k}}^{OPOS,\alpha}\left( \pi_{D} \right)=\bigcup_{i=1}^{m} N_{\sum_{k=1}^{m} A_{k}}^{OPOS,\alpha}\left( D_{i} \right)$ (11)

${DN}_{\sum_{k=1}^{m} A_{k}}^{PPOS,\alpha}\left( \pi_{D} \right)=\bigcap_{i=1}^{m} N_{\sum_{k=1}^{m} A_{k}}^{OPOS,\alpha}\left( D_{i} \right)$ (12)

1. matrix-based approach for NMG-DTRS

Matrix is a commonly used tool in mathematics and has the characteristics of easy representation and calculation, which can improve the computational efficiency in practical application. Therefore, matrix-based techniques play an important role in knowledge representation and data analysis ^[20,25]^. In this subsection, a matrix-based approach was introduced for calculating approximation sets of NMG-DTRS model and the positive, boundary and negative domains were constructed from the matrix perspective in numerical type or hybrid-valued type decision systems.

- 1. Matrix-based decision domains expresentation

**Definition 6.**^[20,25]^ Suppose $NIS=(U,A=C\cup D,V,f)$ be a neighbourhood decision table information system, where U represents the target space objects,$U=\{x_{i}|i=1,2,\cdots,n\}$, for any object subset $X\subseteq U$, order $F(X)$ is the characteristic function of the subset X,$F(X)$ can be constructed by$F\left( X \right)={[f_{i}]}_{n\times1}$, where $F(X)$ is a Boolean vector and $f_{i}$ can be expressed as:

$f_{i}=\left\{ \begin{aligned} 1 , if x_{i}\in X; \\ 0 , if x_{i}\notin X; \end{aligned} \right. i=1,2,\cdots,n.$ (13)

**Definition 7.** Suppose $NIS=(U,A=C\cup D,V,f)$ be a neighbourhood decision table information system, where U represents the target space objects,$U=\{x_{i}|i=1,2,\cdots,n\}$,$A_{1},A_{2},\cdots A_{m}\subseteq A$ are m granular structures, order $M_{A_{k}}={[m_{\mathrm{ij}}^{A_{k}}]}_{n\times n}$ is the neighbourhood relation matrix about granular structure $A_{k}$, where $(k=1,2,\cdots,m)$. For given the neighborhood radius $\delta(\delta\geq0)$ and the metric function$\triangle$, $M_{A_{k}}$ is constructed as:

$m_{ij}^{A_{k}}=\left\{ \begin{aligned} 1 , if \Delta_{A_{k}}\left( x_{i},x_{j} \right)\leq\delta; \\ 0 , otherwise. \end{aligned} \right. (i,j=1,2,\cdots,n).$ (14)

**Definition 8.** For given a neighborhood decision table system$NIS=(U,A=C\cup D,V,f)$, and where U represents the target space objects, $U=\{x_{i}|i=1,2,\cdots,n\}$ , $A_{1},A_{2},\cdots A_{m}\subseteq A$ are m granular structures, for any object subset $X\subseteq U$, suppose $F\left( X \right)={[f_{i}]}_{n\times1}$ is the characteristic function of the subset$X$ and $M_{A_{k}}={[m_{\mathrm{ij}}^{A_{k}}]}_{n\times n}$ is the neighborhood relation matrix on granular structure$A_{k}$, where $(k=1,2,\cdots,m)$. Order $\phi={[1,1,\cdots,1]}^{T}$ denotes an $n\times1$ column vector. Then we can define two intermediate matrices $\overline{H_{A_{k}}(X)}={[\nu_{A_{k}}^{i}]}_{n\times1}$, $\underline{H_{A_{k}}(X)}={[\mu_{A_{k}}^{i}]}_{n\times1}$ and the basic matrix $H_{A_{k}}\left( X \right)={[\omega_{A_{k}}^{i}]}_{n\times1}$ of the subset $X$ about granularity $A_{k}$ as follows:

$\left\{ \begin{aligned} \overline{H_{A_{k}}\left( X \right)}=M_{A_{k}}\times\phi\\ \underline{H_{A_{k}}(X)}=M_{A_{k}}\times F\left( X \right) \\ H_{A_{k}}\left( X \right)=\frac{\underline{H_{A_{k}}(X)}}{\overline{H_{A_{k}}\left( X \right)}} \end{aligned} \right.$ (15)

Where $\mu_{A_{k}}^{i}=\sum_{j=1}^{n} m_{ij}^{A_{k}}\times f_{j},{\nu_{A_{k}}^{i}=\sum_{j=1}^{n} m_{ij}^{A_{k}},\omega}_{A_{k}}^{i}=\mu_{A_{k}}^{i}/\nu_{A_{k}}^{i}(i=1,2,\cdots,n)$, and$"\times"$ indicates matrix multiplication and$"\text{/}\text{"}$ denotes matrix dot divide.

**Definition 9.** For given neighbourhood information system $NIS=(U,A=C\cup D,V,f)$, where U represents the target space objects,$U=\{x_{i}|i=1,2,\cdots,n\}$ $A_{1},A_{2},\cdots A_{m}\subseteq A$ are m granular structures, for any object subset $X\subseteq U$, suppose $H_{A_{k}}\left( X \right)={[\omega_{A_{k}}^{i}]}_{n\times1}$ is the basic matrix of the subset $X$ about granularity $A_{k}$, for given the thresholds$(\alpha,\beta)$, then the matrices of the positive domains, boundary domains, and negative domains of the subset $X$ about granularity $A_{k}$: $H_{A_{k}}^{POS}\left( X \right)={[\omega_{A_{k}^{i}}^{\mathrm{pos}}]}_{n\times1}$,$H_{A_{k}}^{BND}\left( X \right)={[\omega_{A_{k}^{i}}^{\mathrm{bnd}}]}_{n\times1}$, and $H_{A_{k}}^{NEG}\left( X \right)={[\omega_{A_{k}^{i}}^{\mathrm{neg}}]}_{n\times1}$ are defined respectively as follows:

$\omega_{A_{k}^{i}}^{pos}=\left\{ \begin{aligned} 1, if \omega_{A_{k}}^{i}\geq\alpha; \\ 0, otherwise. \end{aligned} \right.$*;*

$\omega_{A_{k}^{i}}^{bnd}=\left\{ \begin{aligned} 1, if {\beta\leq\omega}_{A_{k}}^{i}<\alpha; \\ 0, otherwise. \end{aligned} \right.$ (16)

$\omega_{A_{k}^{i}}^{neg}=\left\{ \begin{aligned} 1,if \omega_{A_{k}}^{i}<\beta; \\ 0, otherwise. \end{aligned} \right.$.

**Definition 10.**^[20]^ For given two matrices ${{B1=[\theta}_{\mathrm{ij}}]}_{n\times n}$ and ${{B2=[\rho}_{\mathrm{ij}}]}_{n\times n}$ , then the minimum and maximum matrices are denoted as respectively:

$${\max\left( B1,B2 \right)=[max(\theta_{ij},\rho_{ij})]}_{n\times n}$$

${\min\left( B1,B2 \right)=[min(\theta_{ij},\rho_{ij})]}_{n\times n}$ (17)

Where $min$ and $max$ operations take the minimum and maximum values.

- 1. The related properties of NMG-DTRS model

Based on the **definition 3, definition 4, definition 9** and the calculation formulas of decision domains of the optimistic and the pessimistic NMG-DTRS model ,the related properties of the neighborhood multigranulation approximation expresses based on matrices can be obtained.

**Theorem 1.** Suppose $NIS=(U,A=C\cup D,V,f)$ be a neighbourhood decision table information system, where U represents the target space objects,$U=\{x_{i}|i=1,2,\cdots,n\}$. Order${\pi_{A}=\{A}_{1},A_{2},\cdots A_{m}\}\subseteq A$ are m granular structures, for given any object subset$X\subseteq U$and the thresholds$(\alpha,\beta)$, order$H_{A_{k}}^{\mathrm{POS}}\left( X \right)={[\omega_{A_{k}^{i}}^{\mathrm{pos}}]}_{n\times1}$,$H_{A_{k}}^{\mathrm{NEG}}\left( X \right)={[\omega_{A_{k}^{i}}^{\mathrm{neg}}]}_{n\times1}$ and $H_{A_{k}}^{\mathrm{BND}}\left( X \right)={[\omega_{A_{k}^{i}}^{\mathrm{bnd}}]}_{n\times1}$denote the matrices of the positive domains, negative domains and boundary domains of the subset $X$ about granularity $A_{k}$ respectively. Then the positive domains, negative domains and boundary domains matrices of the subset $X$ on $\pi_{A}$ based on the optimistic NMG-DTRS model can are obtained as:

$${\left( 1 \right) HN}_{\pi_{A}}^{OPOS,\alpha}\left( X \right)={max}_{k=1}^{m}H_{A_{k}}^{POS}\left( X \right)$$

$${\left( 2 \right) HN}_{\pi_{A}}^{OBND,\alpha\beta}\left( X \right)={min}_{k=1}^{m}H_{A_{k}}^{BND}\left( X \right)$$

$${\left( 3 \right) HN}_{\pi_{A}}^{ONEG,\beta}\left( X \right)={max}_{k=1}^{m}H_{A_{k}}^{NEG}\left( X \right)$$

**Proof.** (1)Denote${{HN}_{\pi_{A}}^{OPOS,\alpha}\left( X \right)=HN}_{\sum_{k=1}^{m} A_{k}}^{OPOS,\alpha}\left( X \right)=[\theta_{1}^{opos}, \theta_{2}^{opos},\cdots,\theta_{n}^{opos}]$and${max}_{k=1}^{m}H_{A_{k}}^{POS}\left( X \right)=[\tau_{1}^{opos}, \tau_{2}^{opos},\cdots,\tau_{n}^{opos}]$. Now suppose the$\theta_{i}^{opos}=1 (\forall i\in\{1,2,\cdots,n\}$*)*, according to formula (5), we have $x_{i}\in N_{\sum_{k=1}^{m} A_{k}}^{OPOS,\alpha}\left( X \right)$,then$\exists k\in\left\{ 1,2,\cdots,m \right\},P_{r}\left( X | N_{A_{k}}\left( x_{i} \right) \right)\geq\alpha$.Thus, for$\forall x_{j}\in N_{A_{k}}\left( x_{i} \right)$ ,we can get $\Delta_{A_{k}}\left( x_{i},x_{j} \right)\leq\delta$. By **Definition 6 and Definition 7**, we can get $m_{ij}^{A_{k}}=1$ and $\omega_{A_{k}}^{i}=(\sum_{j=1}^{n} m_{ij}^{A_{k}}\times f_{j}/\sum_{j=1}^{n} m_{ij}^{A_{k}})\geq\alpha$*.*Namely, according to **Definition 9**, $\exists k\in\left\{ 1,2,\cdots,m \right\}$, $\omega_{A_{k}^{i}}^{pos}=1$. By **Definition 10**,$\tau_{i}^{opos}={max}_{k=1}^{m}{(\omega}_{A_{k}^{i}}^{pos})=1$ holds. Hence, we obtain${HN}_{\sum_{k=1}^{m} A_{k}}^{OPOS,\alpha}\left( X \right)≼{max}_{k=1}^{m}H_{A_{k}}^{POS}\left( X \right)$. When suppose that $\tau_{i}^{opos}=1(\forall i\in\left\{ 1,2,\cdots,n \right\})$, by **Definitions 9 and 10**, necessarily, $\exists k\in\left\{ 1,2,\cdots,m \right\}$*,* $\omega_{A_{k}^{i}}^{pos}=1$. We have $(\sum_{j=1}^{n} m_{ij}^{A_{k}}\times f_{j}/\sum_{j=1}^{n} m_{ij}^{A_{k}})\geq\alpha$, then for any $x_{i}$ and $x_{j}$*(*$i,j\in\{1,2,\cdots,n\})$, $\Delta_{A_{k}}\left( x_{i},x_{j} \right)\leq\delta$, we can get $P_{r}\left( X | N_{A_{k}}\left( x_{i} \right) \right)\geq\alpha$. Thus,$x_{i}\in N_{\sum_{k=1}^{m} A_{k}}^{OPOS,\alpha}\left( X \right)$ holds. Hence, we have $\theta_{i}^{opos}=1$ for given any $\forall i\in\{1,2,\cdots,n\}$. Accordingly, we can obtain ${HN}_{\sum_{k=1}^{m} A_{k}}^{OPOS,\alpha}\left( X \right)≽{max}_{k=1}^{m}H_{A_{k}}^{POS}\left( X \right)$.So, ${HN}_{\pi_{A}}^{OPOS,\alpha}\left( X \right)={max}_{k=1}^{m}H_{A_{k}}^{POS}\left( X \right)$ holds. In the same way, **Theorem 1** ${\left( 2 \right) HN}_{\pi_{A}}^{OBND,\alpha\beta}\left( X \right)={min}_{k=1}^{m}H_{A_{k}}^{BND}\left( X \right)$ and **Theorem 1**${\left( 3 \right) HN}_{\pi_{A}}^{ONEG,\beta}\left( X \right)={max}_{k=1}^{m}H_{A_{k}}^{NEG}\left( X \right)$ are also provable.

**Theorem 2.** Suppose $NIS=(U,A=C\cup D,V,f)$ be a neighbourhood decision table information system, where U represents the target space objects,$U=\{x_{i}|i=1,2,\cdots,n\}$.Order${\pi_{A}=\{A}_{1},A_{2},\cdots A_{m}\}\subseteq A$ are m granular structures, for given any object subset$X\subseteq U$and the thresholds$(\alpha,\beta)$, order$H_{A_{k}}^{\mathrm{POS}}\left( X \right)={[\omega_{A_{k}^{i}}^{\mathrm{pos}}]}_{n\times1}$, $H_{A_{k}}^{\mathrm{BND}}\left( X \right)={[\omega_{A_{k}^{i}}^{\mathrm{bnd}}]}_{n\times1}$, and $H_{A_{k}}^{\mathrm{NEG}}\left( X \right)={[\omega_{A_{k}^{i}}^{\mathrm{neg}}]}_{n\times1}$ denote the matrices of the positive domains, negative domains and boundary domains of the subset $X$ about granularity $A_{k}$ respectively. Then the positive domains, negative domains and boundary domains matrices of the subset $X$ on $\pi_{A}$ based on the pessimistic NMG-DTRS model can are obtained as:

$${\left( 1 \right) HN}_{\pi_{A}}^{PPOS,\alpha}\left( X \right)={min}_{k=1}^{m}H_{A_{k}}^{POS}\left( X \right)$$

$${\left( 2 \right) HN}_{\pi_{A}}^{PBND,\alpha\beta}\left( X \right)={max}_{k=1}^{m}H_{A_{k}}^{BND}\left( X \right)$$

$${\left( 3 \right) HN}_{\pi_{A}}^{PNEG,\beta}\left( X \right)={min}_{k=1}^{m}H_{A_{k}}^{NEG}\left( X \right)$$

**Proof.** For **Theorem2** (1), assume that ${{HN}_{\pi_{A}}^{PPOS,\alpha}\left( X \right)=HN}_{\sum_{k=1}^{m} A_{k}}^{PPOS,\alpha}\left( X \right)={[\theta_{i}^{ppos}]}_{n\times1}$ and${min}_{k=1}^{m}H_{A_{k}}^{POS}\left( X \right)={[\tau_{i}^{ppos}]}_{n\times1}$. Now if the$\theta_{i}^{ppos}=1 (\forall i\in\{1,2,\cdots,n\}$*)*, according to formula (8), we have$x_{i}\in N_{\sum_{k=1}^{m} A_{k}}^{PPOS,\alpha}\left( X \right)$, then for any $k\in\left\{ 1,2,\cdots,m \right\},P\left( X | N_{A_{k}}\left( x_{i} \right) \right)\geq\alpha$. Thus, for $\forall x_{j}\in N_{A_{k}}\left( x_{i} \right)$, we can get$\Delta_{A_{k}}\left( x_{i},x_{j} \right)\leq\delta$. By **Definitions 6 and 7**, for any $k\in\left\{ 1,2,\cdots,m \right\}$ we can get $m_{ij}^{A_{k}}=1$ and$\omega_{A_{k}}^{i}=(\sum_{j=1}^{n} m_{ij}^{A_{k}}\times f_{j}/\sum_{j=1}^{n} m_{ij}^{A_{k}})\geq\alpha$. Namely, according to **Definition 9**,$\forall k\in\left\{ 1,2,\cdots,m \right\}$, $\omega_{A_{k}^{i}}^{pos}=1$*.* Thus, by **Definition 10**,$\tau_{i}^{ppos}={min}_{k=1}^{m}{(\omega}_{A_{k}^{i}}^{pos})=1$ holds. Hence, we obtain ${HN}_{\sum_{k=1}^{m} A_{k}}^{PPOS,\alpha}\left( X \right)≼{min}_{k=1}^{m}H_{A_{k}}^{POS}\left( X \right)$. When assume that $\tau_{i}^{opos}=1$, by **Definitions 8 and 9**, necessarily, for any$k\in\left\{ 1,2,\cdots,m \right\}$, $\omega_{A_{k}^{i}}^{pos}=1$. We have $(\sum_{j=1}^{n} m_{ij}^{A_{k}}\times f_{j}/\sum_{j=1}^{n} m_{ij}^{A_{k}})\geq\alpha$, then for any $x_{i}$ and $x_{j}$($i,j\in\{1,2,\cdots,n\})$*,* $\Delta_{A_{k}}\left( x_{i},x_{j} \right)\leq\delta$, we can get $P_{r}\left( X | N_{A_{k}}\left( x_{i} \right) \right)\geq\alpha$. Then, $x_{i}\in N_{\sum_{k=1}^{m} A_{k}}^{PPOS,\alpha}\left( X \right)$ holds. Hence, we have $\theta_{i}^{ppos}=1$ for given any $\forall i\in\{1,2,\cdots,n\}$. Accordingly, we can get ${HN}_{\sum_{k=1}^{m} A_{k}}^{PPOS,\alpha}\left( X \right)≽{min}_{k=1}^{m}H_{A_{k}}^{POS}\left( X \right)$. So,${HN}_{\sum_{k=1}^{m} A_{k}}^{PPOS,\alpha}\left( X \right)={min}_{k=1}^{m}H_{A_{k}}^{POS}\left( X \right)$, namely${HN}_{\pi_{A}}^{PPOS,\alpha}\left( X \right)={min}_{k=1}^{m}H_{A_{k}}^{POS}\left( X \right)$*.* For **Theorem 2** (2) and (3), the proof is similar to **Theorem 2** (1), they will be omitted.

The following lemma regarding the positive domain matrices of the optimistic and the pessimistic NMG-DTRS models can be obtained.

**Lemma 1.** Given neighbourhood decision table information system$NIS=(U,A=C\cup D,V,f)$, where U represents the target space objects,$U=\{x_{i}|i=1,2,\cdots,n\}$.Order${\pi_{A}=\{A}_{1},A_{2},\cdots A_{m}\}\subseteq A$ are m granular structures, for the given thresholds$0.5\leq\alpha_{1}<\alpha_{2}\leq1$ and two object subset $\forall X\subseteq\forall Y\subseteq U$, then we can get the following properties:

$${\left( 1 \right) HN}_{\pi_{A}}^{OPOS,\alpha_{1}}\left( X \right){≼ HN}_{\pi_{A}}^{OPOS,\alpha_{1}}\left( Y \right)$$

$${\left( 2 \right) HN}_{\pi_{A}}^{PPOS,\alpha_{1}}\left( X \right){≼ HN}_{\pi_{A}}^{PPOS,\alpha_{1}}\left( Y \right)$$

$${\left( 3 \right) HN}_{\pi_{A}}^{OPOS,\alpha_{1}}\left( X \right){≽ HN}_{\pi_{A}}^{OPOS,\alpha_{2}}\left( X \right)$$

$${\left( 4 \right) HN}_{\pi_{A}}^{PPOS,\alpha_{1}}\left( X \right){≽ HN}_{\pi_{A}}^{PPOS,\alpha_{2}}\left( X \right)$$

The relevant proof is omitted.

**Lemma 2.** Given neighbourhood decision table information system$NIS=(U,A=C\cup D,V,f)$, and where U represents the target space objects,$U=\{x_{i}|i=1,2,\cdots,n\}$.Order${\pi_{A}=\{A}_{1},A_{2},\cdots A_{m}\}\subseteq A$ are m granular structures, suppose ${A1}^{'}\subseteq A1$, for given the thresholds$(\alpha,\beta)$ ,and for any the object subset $X\subseteq U$ ,then the following related properties can be obtained:

$${\left( 1 \right) HN}_{\sum_{A_{k}\in A1} A_{k}}^{OPOS,\alpha}\left( X \right){≽ HN}_{\sum_{A_{k}\in{A1}^{'}} A_{k}}^{OPOS,\alpha}\left( X \right)$$

$${\left( 2 \right) HN}_{\sum_{A_{k}\in A1} A_{k}}^{PPOS,\alpha}\left( X \right){≼ HN}_{\sum_{A_{k}\in{A1}^{'}} A_{k}}^{PPOS,\alpha}\left( X \right)$$

**Proof**. (1) denote ${{HN}_{\sum_{A_{k}\in A1} A_{k}}^{OPOS,\alpha}\left( X \right)=HN}_{\sum_{k=1}^{m} A_{k}}^{OPOS,\alpha}\left( X \right)=[\theta_{1}^{opos}, \theta_{2}^{opos},\cdots,\theta_{n}^{opos}]$and${HN}_{\sum_{A_{k}\in{A1}^{'}} A_{k}}^{OPOS,\alpha}\left( X \right)=[\tau_{1}^{opos}, \tau_{2}^{opos},\cdots,\tau_{n}^{opos}]$. According to **Theorem 1** (1) , we have ${{HN}_{\sum_{A_{k}\in A1} A_{k}}^{OPOS,\alpha}\left( X \right)=HN}_{\pi_{A}}^{OPOS,\alpha}\left( X \right)={max}_{k=1}^{m}H_{A_{k}}^{POS}\left( X \right)$, for $\forall\theta_{i}^{opos}\in{HN}_{\sum_{A_{k}\in A1} A_{k}}^{OPOS,\alpha}\left( X \right)$, order $H_{A_{k}}^{POS}\left( X \right)={[\omega_{A_{k}^{i}}^{pos}]}_{n\times1}$ , by **Definition 10**, we have $\theta_{i}^{opos}=max(\left[ \omega_{A_{1}^{i}}^{pos} \right]_{n\times1},\left[ \omega_{A_{2}^{i}}^{pos} \right]_{n\times1},\cdots,\left[ \omega_{A_{m}^{i}}^{pos} \right]_{n\times1})$. When${A1}^{'}\subseteq A1$, $\mathrm{suppose} \exists A_{p}\notin{A1}^{'}\mathrm{and}A_{p}\in A1$, then $\forall\tau_{i}^{opos}\in{HN}_{\sum_{A_{k}\in{A1}^{'}} A_{k}}^{OPOS,\alpha}\left( X \right)=max(\left[ \omega_{A_{1}^{i}}^{pos} \right]_{n\times1},\cdots,\left[ \omega_{A_{p-1}^{i}}^{pos} \right]_{n\times1},\left[ \omega_{A_{p+1}^{i}}^{pos} \right]_{n\times1},\cdots,\left[ \omega_{A_{m}^{i}}^{pos} \right]_{n\times1})$, for any k, if have$\left[ \omega_{A_{p}^{i}}^{pos} \right]_{n\times1}>\left[ \omega_{A_{k}^{i}}^{pos} \right]_{n\times1}(k\neq p)$ , then $\theta_{i}^{opos}>\tau_{i}^{opos}$, otherwise $\theta_{i}^{opos}=\tau_{i}^{opos}$. Namely ${HN}_{\sum_{A_{k}\in A1} A_{k}}^{OPOS,\alpha}\left( X \right){≽ HN}_{\sum_{A_{k}\in{A1}^{'}} A_{k}}^{OPOS,\alpha}\left( X \right)$ holds. In the same way, Lemma **2** (2) ${HN}_{\sum_{A_{k}\in A1} A_{k}}^{PPOS,\alpha}\left( X \right){≼ HN}_{\sum_{A_{k}\in{A1}^{'}} A_{k}}^{PPOS,\alpha}\left( X \right)$is also provable.

- 1. Algorithm implementation for solving decision domains in NMG-DTRS

**Definition 11.** Given neighbourhood decision table information system$NIS=(U,A=C\cup D,V,f)$, and where U represents the target space objects, D denotes decision attribute, $\pi_{A}=\{A_{1},A_{2},\cdots A_{m}\}\subseteq A$ are m granular structures, for given the pair of thresholds$(\alpha,\beta)$ and any object subset $X\subseteq U$,order${HN}_{\pi_{A}}^{OPOS,\alpha}\left( X \right)$,${HN}_{\pi_{A}}^{ONEG,\beta}\left( X \right)$and ${HN}_{\pi_{A}}^{OBND,\alpha\beta}\left( X \right)$are the matrices of the positive domains, negative domains and boundary domains of the subset $X$ about granularity structure $\pi_{A}$ based on the optimistic NMG-DTRS model and ${HN}_{\pi_{A}}^{PPOS,\alpha}\left( X \right)$,${HN}_{\pi_{A}}^{PNEG,\beta}\left( X \right)$and ${HN}_{\pi_{A}}^{PBND,\alpha\beta}\left( X \right)$ are the matrices of the positive domains, negative domains and boundary domains of the subset $X$ about granularity structure $\pi_{A}$ based on the pessimistic NMG-DTRS model respectively. Suppose $\pi_{D}=\{D_{1},D_{2},\cdots,D_{m}\}$ are the partitions on the target space objects $U$ about decision attribute$D$, then the optimistic positive domain, boundary domain, and negative decision domain matrices based on $\pi_{D}$ in NMG-DTRS model can be defined by:

$${DHN}_{\pi_{A}}^{OPOS,\alpha}\left( \pi_{D} \right)=\{\bigcup_{i=1}^{m} {HN}_{\pi_{A}}^{OPOS,\alpha}\left( D_{i} \right)\}$$

$${DHN}_{\pi_{A}}^{OBND,\alpha\beta}\left( \pi_{D} \right)=\{\bigcup_{i=1}^{m} {HN}_{\pi_{A}}^{OBND,\alpha\beta}\left( D_{i} \right)\}$$

$${DHN}_{\pi_{A}}^{ONEG,\beta}\left( \pi_{D} \right)=\{\bigcup_{i=1}^{m} {HN}_{\pi_{A}}^{ONEG,\beta}\left( D_{i} \right)\}$$

and the pessimistic positive domain, boundary domain and negative decision domain matrices based on $\pi_{D}$ in NMG-DTRS model can be defined by:

$${DHN}_{\pi_{A}}^{PPOS,\alpha}\left( \pi_{D} \right)=\{\bigcup_{i=1}^{m} {HN}_{\pi_{A}}^{PPOS,\alpha}\left( D_{i} \right)\}$$

$${DHN}_{\pi_{A}}^{PBND,\alpha\beta}\left( \pi_{D} \right)=\{\bigcup_{i=1}^{m} {HN}_{\pi_{A}}^{PBND,\alpha\beta}\left( D_{i} \right)\}$$

$${DHN}_{\pi_{A}}^{PNEG,\beta}\left( \pi_{D} \right)=\{\bigcup_{i=1}^{m} {HN}_{\pi_{A}}^{PNEG,\beta}\left( D_{i} \right)\}$$

Where $\cup$ denotes the disjunction(OR) operations of matrices.

**Definition 12.** Positive decision recognition rate. Given $NIS=(U,A=CUD,V,f)$ be neighborhood decision information system, where $\pi_{A}=\{A_{1},A_{2},\cdots A_{m}\}\subseteq A$ are m granular structures, D is decision attribute and $\pi_{D}=\{D_{1},D_{2},\cdots,D_{n}\}$ are the partitions on the target space objects $U$ about decision attribute$D$. Order${DHN}_{\pi_{A}}^{POS,\alpha}\left( \pi_{D} \right)$ is the matrix of the positive regions of $\pi_{D}$ with respect to $\pi_{A}$, then Positive decision recognition rate in NMG-DTRS model can be defined by:

$$RecRate=\frac{|{DHN}_{\pi_{A}}^{POS,\alpha}\left( \pi_{D} \right)|}{\sum_{i=1}^{n} |D_{i}|}$$

Where $|{DHN}_{\pi_{A}}^{POS,\alpha}\left( \pi_{D} \right)|$ denotes the number of elements with a value of “1” in the positive decision matrices. The definition of $RecRate$ reflects the probability of correctly identifying positive decision under granularity $\pi_{A}$ for the decision table neighborhood information system.

According to **Definition 11** and **Definition 12**, Matrix-based algorithms for decision regions in NMG-DTRS can be described as algorithm 1, algorithm 2 and algorithm 3 in Fig. 1 to Fig. 3.

In Algorithm 1, suppose number of attributes is |C|, for each granularity $A_{i}\in A^{'},$ the calculation of the neighbourhood relation matrix and the basic matrix have to be executed, namely the maximum time complexity computing the neighbourhood relation matrix is $O(|C|\cdot{|U|}^{2})$, and the time complexity of calculating the decision domain matrices under the granularity $A_{i}$ by Algorithm 2 is $O({|U|}^{2})$, so, the time complexity of step 6-9 in Algorithm 1 is $O(m\cdot|C|\cdot{|U|}^{2})$, and the time consumption for executing the repeat loop operation in step 10-12 is $O({|U|}^{2})$. Therefore, for all decision partition objects$U/{D=\{D_{1},D_{2},\cdots,D_{n}\}}$, the total time complexity of **Algorithm 1** is $O(mn\cdot|C|\cdot{|U|}^{2})$.


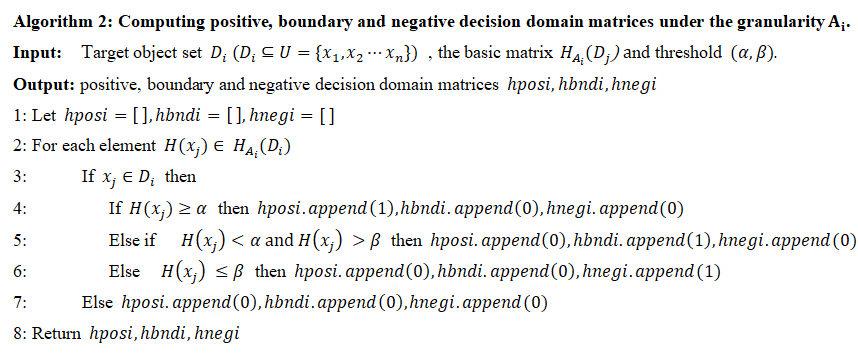


**Fig. 2** **Computing decision domain matrices**


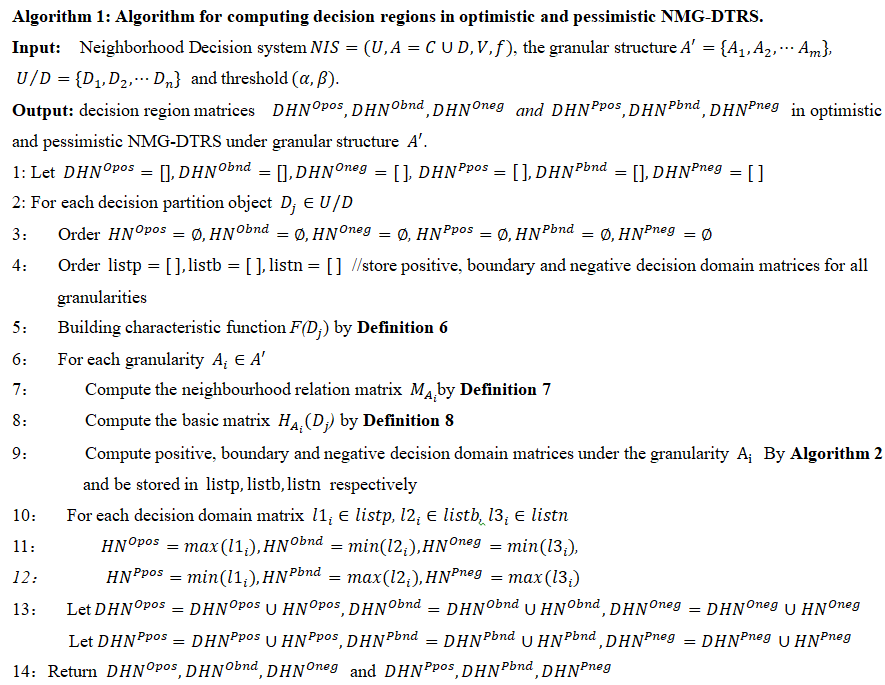


**Fig. 1** **Matrix-based algorithm for decision regions**

${}$ In Algorithm 3, the positive decision region matrices $\mathrm{DHN}^{\mathrm{Opos}}$and $\mathrm{DHN}^{\mathrm{Ppos}}$ be firstly executed in view of Algorithm 1, we can get the time complexity is $O(mn\cdot|C|\cdot{|U|}^{2})$, and the time complexity of step 3-5 is $O(|U|)$.Thus, the total time complexity in Algorithm 3 is also $O(mn\cdot|C|\cdot{|U|}^{2})$.


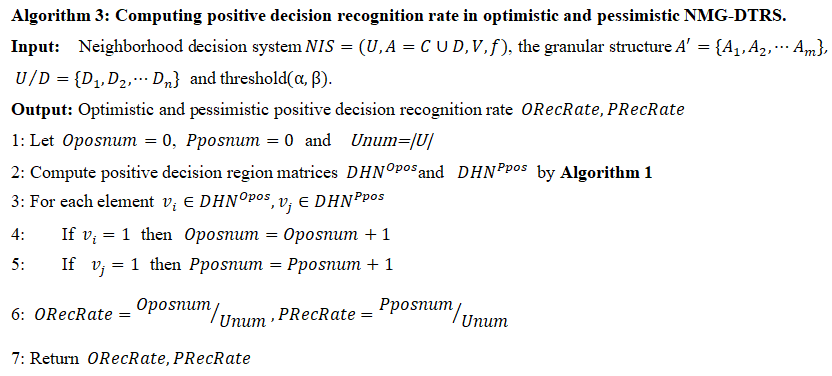


**Fig. 3 Solving positive decision recognition rate**

1. Experimental analysis


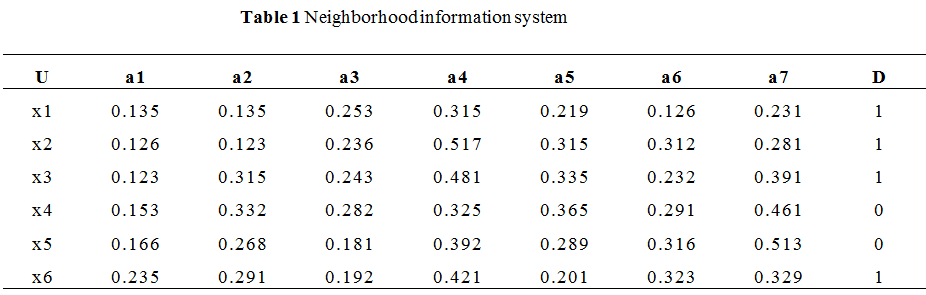
 For given a decision table neighborhood information system NIS, which was shown in **Table 1**, and where ${U=\{x}_{1},x_{2},\cdots{,x}_{6}\}$indicate the objects of decision system, ${A=\{a}_{1},a_{2},\cdots,a_{7}\}$ denotes attributes, D is decision attribute, and decision partition object sets on the universe $U$ based on decision attribute $D$ are ${\{x}_{1},x_{2},x_{3}{,x}_{6}\}$ and $\{x_{4},x_{5}\}$. In the process of experiment, assumed the neighborhood radius $\delta=0.15$, and three different granularity spaces were selected for experiment when the threshold $(\alpha,\beta)$ is (1,0), (0.8,0.2) and (0.65,0.3) respectively. All experimental results were gained from **table 1** in python3.6 programming environment, and the experimental results were shown in **Table 2** and **Table 3(**see Appendix 1 for **Table 2** and **Table 3)**. For convenience of expression, in the **Table 2,** OPOS,OBND and ONEG denote positive, boundary and negative decision domain matrices obtained in optimistic NMG-DTRS, and ODPOS and ORecRate denote positive decision matrices and positive decision recognition rate for neighborhood decision information system in optimistic NMG-DTRS model, at the same time, in the **Table 3**, PPOS,PBND and PNEG denote positive, boundary and negative decision domain matrices obtained in pessimistic NMG-DTRS, and PDPOS and PRecRate denote positive decision matrices and positive decision recognition rate for neighborhood decision information system in pessimistic NMG-DTRS model.


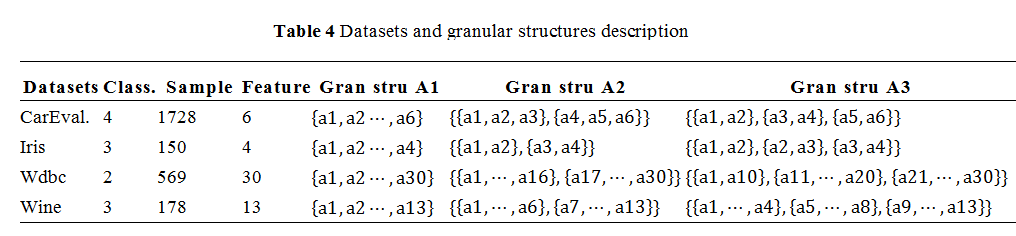
The experimental results in Table 2 and Table 3 show that the method based on matrices proposed in this paper can effectively calculate the decision domains of neighborhood decision system, and it can also be seen that with the change of threshold and granularity, the decision domains and decision recognition rate also change. At the same time, it can be analyzed in the light of information in the Table 2 and Table 3 that the positive decision recognition rate reflects the importance of granularity; on the other hand, the decrease of positive decision is mainly caused by the increase of boundary domain. Therefore, in the actual decision-making process, we can make a decision again for the instance in boundary domain, so as to increase the reliability of decision-making.

In order to further verify the effectiveness and feasibility of the approach, several standard datasets provided in UCI database were selected as experimental objects, and the specific description of datasets was shown in **Table 4**. In the process of experiments on four different datasets, under the condition of a certain threshold, the positive decision recognition rates obtained from the optimistic and pessimistic NMG-DTRS models were analyzed and compared from the perspective of different granularity. See Appendix 2 for Fig.4 (a)-(f) and Appendix 2 for Fig.5 (g)-(l). Through the experimental results of four datasets, we can see that the positive decision recognition rate under different granularity is quite different when a certain threshold is given, and the positive decision recognition rate under the optimistic NMG-DTRS is always greater than or equal to that under the pessimistic NMG-DTRS. At the same time, the relationship between decision domain and threshold was analyzed, as shown in Fig.6(Appendix 2 for Fig.6). According to g(1), g(3) and g(5) in Fig.6, it can be seen that under certain granularity, the change of positive decision recognition rate with threshold is not obvious in optimistic NMG-DTRS, while the positive decision recognition rate in pessimistic NMG-DTRS is relatively influenced by threshold and granularity. See g(2), g(4) and g(6) in Fig. 6.According to the experimental results, we get that the importance of granularity is different, and the contribution to decision-making is also different, so the positive decision recognition rate obtained under different granularity is different. Therefore, when we make decision on complex hybrid information system, we can make decision analysis from multiple perspectives, and at the same time, we can select appropriate threshold to limit the decision decision-making conditions, so as to avoid the phenomenon of one-time decision-making leading to misjudgement and improve the reliability of decision-making.

1. Conclusion

MG-DTRS models can analyze decision-making problems from multi-level and multi-angle, the neighbourhood rough set model has the advantage of processing numerical or hybrid-valued data, NMG-DTRS models effectively integrate MG-DTRS and NRS models, greatly expanding the application range of MG-DTRS. The paper proposed a novel matrix-based method for approximation sets of NMG-DTRS model and solved the decison domains through a series of matrix calculation, the related properties of NMG-DTRS model were explored and the implementation algorithms based on the proposed method were designed and discussed in detail. The research of NMG-DTRS models matrix-based in this paper makes use of the characteristics of matrix which is easy to calculate and understand, and fully combines the advantages of matrix and MG-DTRS, which effectively solves the decision-making problems of complex decision information systems from another aspect and expands the application scope of NMG-DTRS. The use of matrix-based NMG-DTRS models for solving the decision-making problems in big data and dynamic data context will be the target of the further research.

1. Acknowledgments

The authors of this paper sincerely acknowledge the support of the Universities Natural Science Key Project of Anhui Province (No.KJ2020A0637).

1. References
2. Z Pawlak. [Rough sets](http://link.springer.com/article/10.1007/BF01001956) [J]. [International Journal of Computer & Information Sciences](http://link.springer.com/journal/10766) 1982, 11(5)：341-356.
3. [M Kryszkiewicz](https://xueshu.glgoo.org/citations?user=HW1AIjQAAAAJ&hl=zh-CN&oi=sra). [Rough set approach to incomplete information systems](http://www.sciencedirect.com/science/article/pii/S0020025598100191)[J]. [Information Sciences](http://www.sciencedirect.com/science/journal/00200255),[Volume 112, Issues 1-4](http://www.sciencedirect.com/science/journal/00200255/112/1), December 1998, Pages 39-49.
4. MIAO Duoqian, LI Daoguo. Rough set theory, algorithm and application[M]. Beijing: Science Press,2007.
5. Yiyu Yao. [Decision-theoretic rough set models](http://link.springer.com/chapter/10.1007/978-3-540-72458-2_1) [J].[Rough Sets and Knowledge Technology](http://link.springer.com/book/10.1007/978-3-540-72458-2), 2007, 4481:1-12.
6. Yuhua Qian, Hu Zhang, Yanli Sang, Jiye Liang. Multigranulation decision-theoretic rough sets[J]. international Journal of Approximate Reasoning, 55 (2014): 225-237.
7. Yang, HL. & Guo, ZL. Multigranulation decision-theoretic rough sets in incomplete information systems[J]. [International Journal of Machine Learning and Cybernetics](https://link.springer.com/journal/13042), 2015, 6(6):1005–1018.
8. [Hengrong Ju](http://www.sciencedirect.com/science/article/pii/S095070511730062X), [Huaxiong Li](http://www.sciencedirect.com/science/article/pii/S095070511730062X),[Xibei Yang](http://www.sciencedirect.com/science/article/pii/S095070511730062X), [Xianzhong Zhou](http://www.sciencedirect.com/science/article/pii/S095070511730062X).Cost-sensitive rough set: A multi-granulation approach[J],[Knowledge-Based Systems](http://www.sciencedirect.com/science/journal/09507051),2017,123(1): 137-153.
9. Jiajun Chen,Yuanyuan Huang, Wenjie Wei et al.Granularity Reduction Method Based on Positive Decision Holding for Multi-granulation Decision-Theoretic Rough Set[J], The Journal of Engineering, 2018, 2018(10): 1389-1395.
10. Huaxiong Li, Xianzhong Zhou. [Risk decision making based on decision-theoretic rough set: a three-way view decision model](http://www.tandfonline.com/doi/abs/10.1080/18756891.2011.9727759)[J].International Journal of Computational Intelligence Systems,2011,4(1):1-11.
11. [Huili Dou](http://www.sciencedirect.com/science/article/pii/S0950705115003500), [Xibei Yang](http://www.sciencedirect.com/science/article/pii/S0950705115003500), [Xiaoning Song](http://www.sciencedirect.com/science/article/pii/S0950705115003500), [Hualong Yu](http://www.sciencedirect.com/science/article/pii/S0950705115003500),[Wei-Zhi Wu](http://www.sciencedirect.com/science/article/pii/S0950705115003500). [Jingyu Yang](http://www.sciencedirect.com/science/article/pii/S0950705115003500).[Decision-theoretic rough set: a multicost strategy](http://www.sciencedirect.com/science/article/pii/S0950705115003500) [J].[Knowledge-Based Systems](http://www.sciencedirect.com/science/journal/09507051), 2016[( 91](http://www.sciencedirect.com/science/journal/09507051/91/supp/C)): 71-83.
12. Mingliang Suo, Laifa Tao, Baolong Zhu, Xuewen Miao, Zhichao Liang, Yu Ding, Xingliu Zhang, Tong Zhang.Single-parameter decision-theoretic rough set[J],Information Sciences, 2020(539): 49-80.
13. T. Yin, X. Mao, Y. Zhang, Y. Ma, H. Ju and W. Ding. Decision-Theoretic Rough Set: A Fusion Strategy[8], IEEE Access, 2020(8): 221027- 221038.
14. Yanting Guo, Eric C.C. Tsang, Weihua Xu, Degang Chen.Adaptive weighted generalized multi-granulation interval-valued decision-theoretic rough sets[J],Knowledge-Based Systems,2020(187):104804.
15. Jiajun Chen , Shuhao Yu, Wenjie Wei , Zhongrong Shi. Incomplete neighbourhood multi-granulation decision-theoretic rough set in the hybrid valued decision system[J], The Journal of Engineering, 2019, 2019 (12):8477-8488.
16. Yuhua Qian, Hu Zhang, Yanli Sang, Jiye Liang. multi-granulation decision-theoretic rough sets[J]. international Journal of Approximate Reasoning, 55 (2014): 225-237.
17. Lin T Y.Neighborhood systems and approximation in database and knowledge base systems[C].Proceedings of the Fourth International Symposium on Methodologies of Intelligent Systems，Poster Session，1989: 75-86．
18. Lin T Y． Granular and nearest neighborhoods: rough set approach[J].Studies in Fuzziness &Soft Computing,2001,70 (15) :125-142．
19. Li Weiwei, Huang Zhiqiu, Jia Xiuyi,et al. Neighborhood based decision-theoretic rough set models[J]. International Journal of Approximate Reasoning, 2016, 69(C): 1-17.
20. Lin Sun, Lanying Wang, Weiping Ding, Yuhua Qian, Jiucheng Xu.Neighborhood multi-granulation rough sets-based attribute reduction using Lebesgue and entropy measures in incomplete neighborhood decision systems[J],Knowledge-Based Systems,2020(192):105373.
21. Chengxiang Hu ,Li Zhang.A dynamic framework for updating neighborhood multigranulation approximations with the variation of objects[J]. Information Sciences ,2020(519): 382–406.
22. ] J.B. Zhang, T.R. Li, D. Ruan, D. Liu.Rough sets based matrix approaches with dynamic attribute variation in set-valued information systems[J],Approximate Reasoning, 2012, 53 (4) :620–635.
23. Chuan Luo,Tianrui Li,Zhang Yi ,Hamido Fujita.Matrix approach to decision-theoretic rough sets for evolving data[J],Knowledge-Based Systems, 2016(99): 123-134.
24. X. Hu , S.X. Liu , G.X. Liu , Matrix-based approaches for dynamic updating approximations in multigranulation rough sets[J],Knowledge-Based Systems, 2017(122) :51–63 .
25. J. Zhang, J. Wong, Y. Pan and T. Li. A Parallel Matrix-Based Method for Computing Approximations in Incomplete Information Systems[J] ,IEEE Transactions on Knowledge and Data Engineering, 2015(27):326-339.
26. [Yu Peiqiu](https://content.iospress.com/search?q=author%3A%28%22Yu,%20Peiqiu%22%29),[Wang Hongkun](https://content.iospress.com/search?q=author%3A%28%22Wang,%20Hongkun%22%29), [Li Jinjin](https://content.iospress.com/search?q=author%3A%28%22Li,%20Jinjin%22%29),[Lin Guoping](https://content.iospress.com/search?q=author%3A%28%22Lin,%20Guoping%22%29). Matrix-based approaches for updating approximations in neighborhood multigranulation rough sets while neighborhood classes decreasing or increasing[J], [Journal of Intelligent & Fuzzy Systems](https://content.iospress.com/journals/journal-of-intelligent-and-fuzzy-systems), 2019, 37(2): 2847-2867.
27. [CaihuiLiu,KecanCai,DuoqianMiao,JinQian.](https://www.sciencedirect.com/science/article/pii/S0020025520306095#!)Novel matrix-based approaches to computing minimal and maximal descriptions in covering-based rough sets[J]. [Information Sciences](https://www.sciencedirect.com/science/journal/00200255), 2020(539): 312-326
28. Alcantud, José Carlos R., Jianming Zhan. Multi-granular soft rough covering sets[J].Soft Compute, 2020(24):9391-9402.
29. Xueling Ma, Jianming Zhan , Bingzhen Sun et al.Novel classes of coverings based multigranulation fuzzy rough sets and corresponding applications to multiple attribute group decision-making[J]. Artificial Intelligence Review,2020 (53):6197-6256.
30. Appendices

Appendix 1
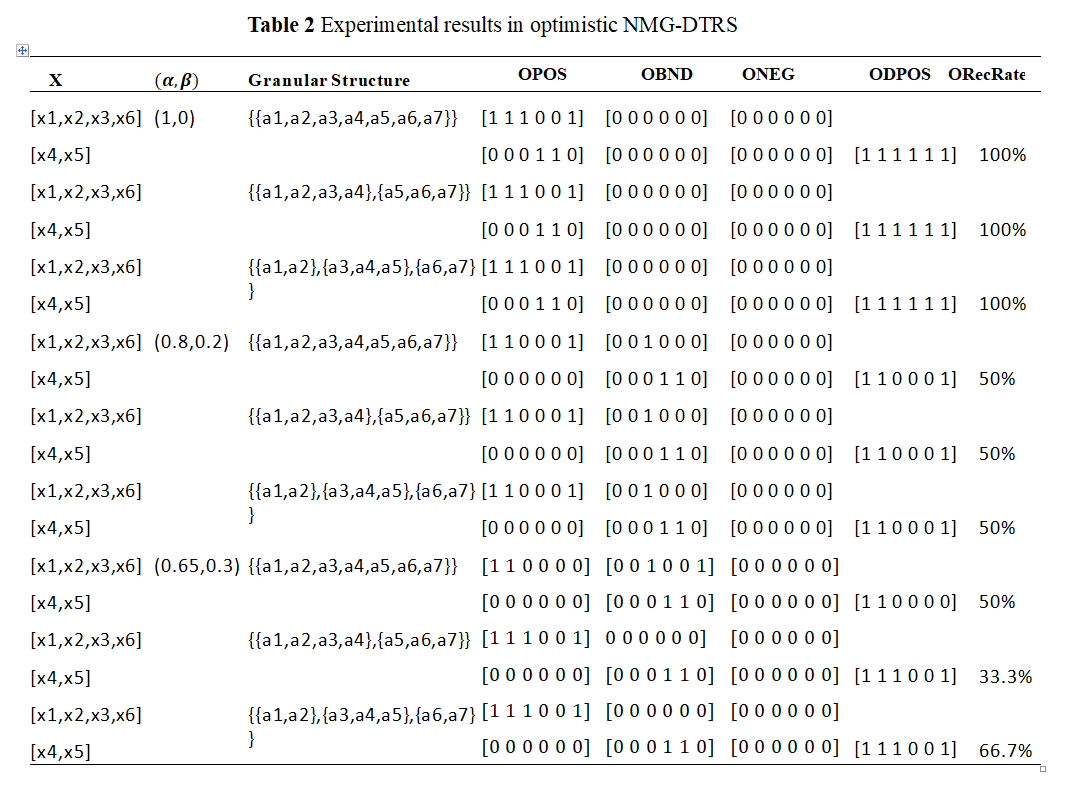


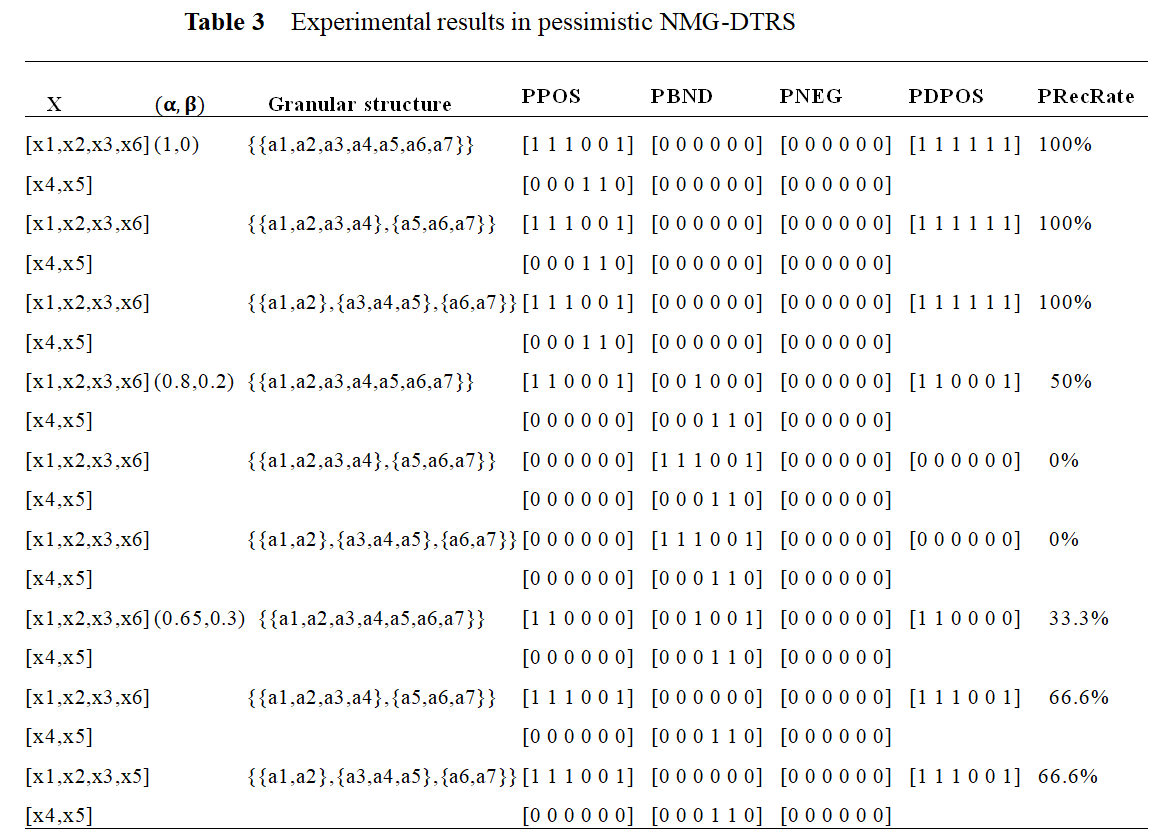


Appendix 2


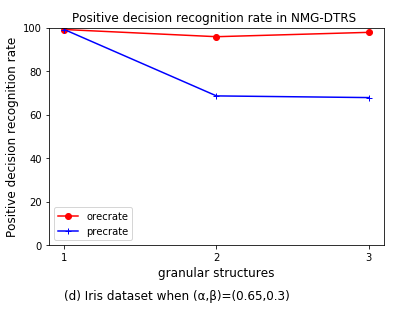

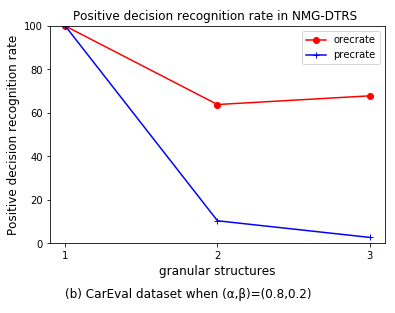

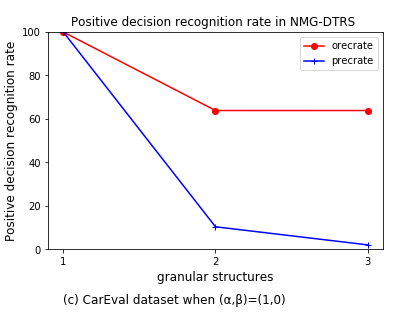

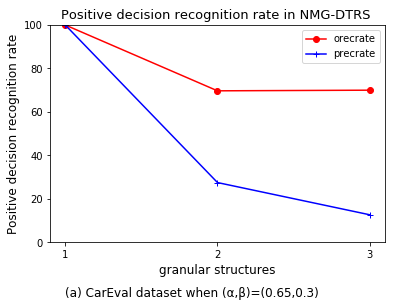

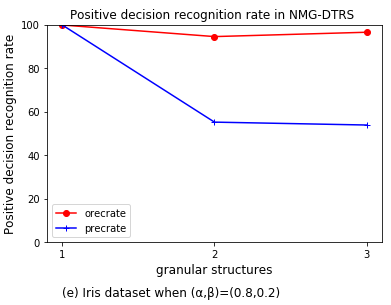

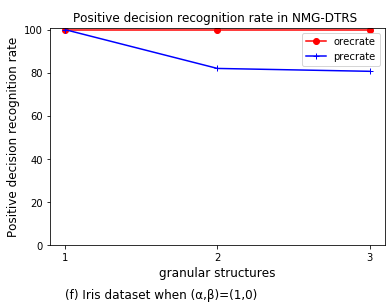


**Fig. 4** *Comparison of decision recognition rate in optimistic and pessimistic NMG-DTRS*


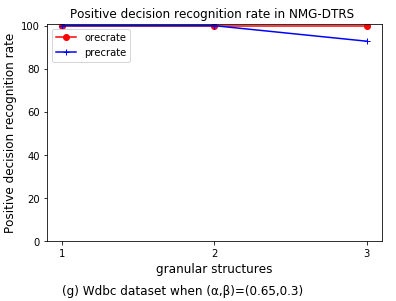

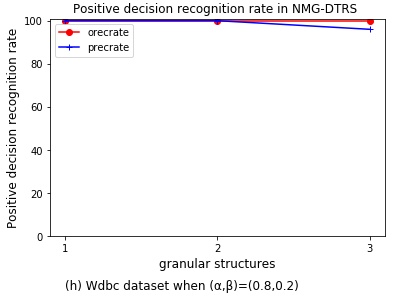

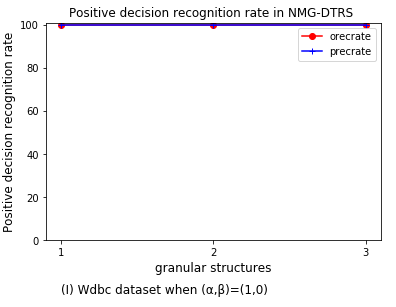

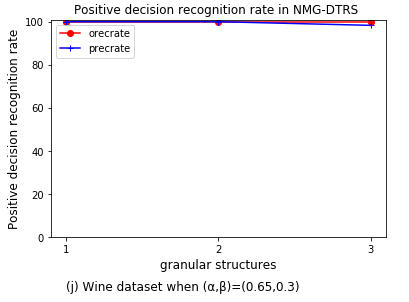

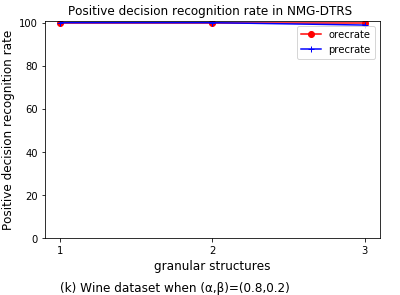

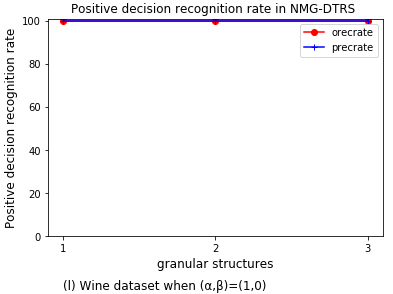


**Fig. 5** *Comparison of decision recognition rate in optimistic and pessimistic NMG-DTRS*


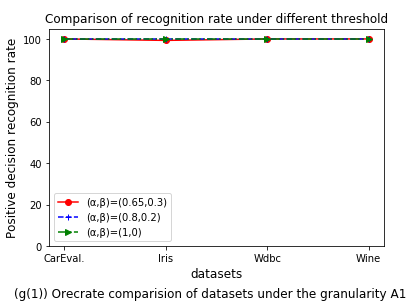

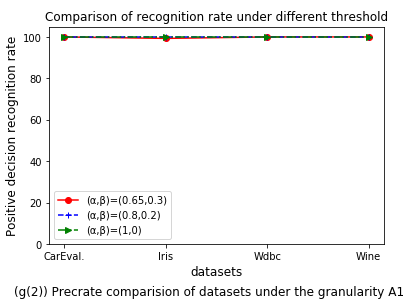

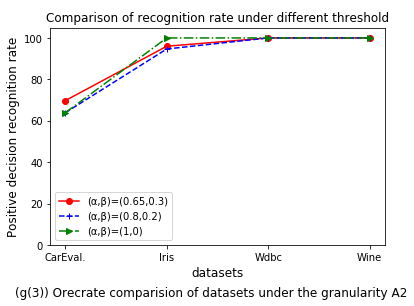

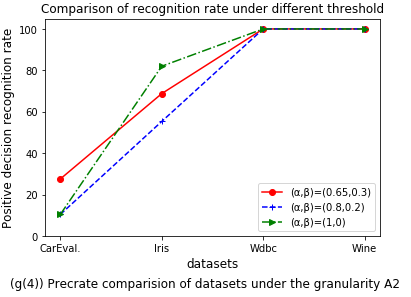

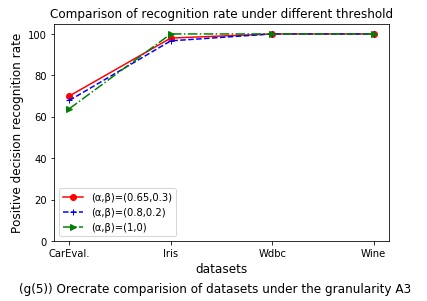

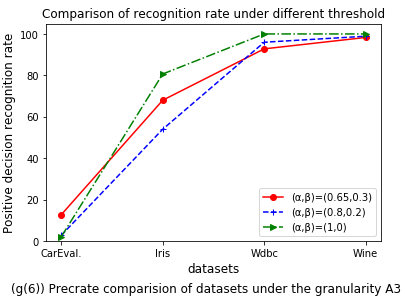


**Fig. 6** *Comparison of decision recognition rate based on different threshold*
